# Meeting abstracts from International Workshop on Pharmaceutical and Clinical Research 2025 (WPCR2025)

**DOI:** 10.1097/MD.0000000000042672

**Published:** 2025-06-06

## 001 | Effect of dexamethasone as an adjuvant in brachial plexus block on postoperative outcome

Liping He^1^, Zhiqiang Fu^1^, Yuechun Lu^2^

^*1*^*Department of Anesthesiology, Tianjin Fifth Central Hospital of Ecological City Hospital, Tianjin, China,* ^*2*^*Department of Anesthesiology, Tianjin Medical University Second Hospital, Tianjin, China*

**Objective:** The purpose is to clarify the effect of dexamethasone as an adjuvant for brachial plexus block on the postoperative outcome of patients and to help optimize the clinical plan.

**Methods:** 180 patients undergoing upper limb surgery who required brachial plexus block anesthesia from January 2022 to December 2024 were selected and randomly divided into two groups. The control group received a conventional brachial plexus block with 0.5% ropivacaine, and the observation group received 5 mg dexamethasone on this basis. The onset and duration of sensory and motor block, 24-hour postoperative visual analogue scale (VAS) pain score, and adverse reactions were recorded in the two groups. SPSS 22.0 software was used for analysis, and the measurement data were expressed as mean ± standard deviation, and the independent sample t test was performed, the counting data were expressed as a rate (%), and the χ² test was used, and P<0.05 was considered statistically significant.

**Results:** The onset time of sensory and motor block in the observation group was (6.2±1.5) min and (8.5±2.1) min, respectively, which was shorter than (8.9±2.3) min and (11.4±2.8) min in the control group (P<0.05), the duration of block was (230.5±35.2) min and (210.8±30.6) min, respectively, which was longer than (185.3±32.1) min and (160.4±28.5) min in the control group (P<0.05). Twenty-four hours after surgery, the VAS pain score of the observation group was (3.1±0.8) points, which was lower than (4.3±1.2) points in the control group (P<0.05). There was no significant difference in the incidence of adverse reactions between the two groups (P>0.05).

**Conclusion:** Dexamethasone as an adjuvant can improve the effect of brachial plexus block, shorten the onset time, prolong the block duration, and relieve postoperative pain without increasing adverse reactions. It is beneficial to postoperative outcomes and is worthy of clinical application.

## 002 | The anti-gastric cancer effect and mechanism of Zuojin Pills based on network pharmacology and miR-107

Rui Liu^1#^, Yuepeng Qiao^2#^, Ying Wang^3^, Yaping Jiang^4^

^*1*^*Department of Traditional Chinese Medicine, Heilongjiang University of Chinese Medicine, Harbin, China,* ^*2*^*Traditional Chinese Medicine Oncology Department Harbin Jiarun Hospital, Harbin, China,* ^*3*^*Department of Traditional Chinese Medicine, The First Affiliated Hospital of Heilongjiang University of Traditional Chinese Medicine, Harbin, China,* ^*4*^*Department of Pharmacy of Universal Xi’an west airlines Hospital, Xi’an, China.* ^*#*^*These authors contribute equally to this study.*

**Objective:** The objective is to analyze the anti-gastric cancer effect and potential mechanism of Zuojin Pills by using network pharmacology combined with miR-107.

**Methods:** The active ingredients and targets of Zuojin Pills were mined from the Traditional Chinese Medicine System Pharmacology Database (TCMSP) and other databases, and gastric cancer disease targets were obtained from databases such as GeneCards. A protein-protein interaction (PPI) network was constructed using the STRING database, and key targets were screened using Cytoscape software. Gene ontology (GO) and Kyoto Encyclopedia of Genes and Genomes (KEGG) enrichment analysis were performed to predict the mechanism of action. In gastric cancer cell lines, the expression of miR-107 and related targets was detected by real-time fluorescence quantitative PCR (qRT-PCR) and Western blot, and the effect of Zuojin Pills on gastric cancer cells was verified by cell proliferation, migration, and invasion experiments.

**Results:** Network pharmacology screened out 15 active ingredients and 108 potential targets of Zuojin Pills, and 32 key targets were obtained by intersection with gastric cancer targets. GO and KEGG analysis showed that these targets were enriched in cell proliferation, apoptosis, migration, and other processes and PI3K-Akt pathways. Cell experiments showed that Zuojin Pills could significantly inhibit gastric cancer cell proliferation, migration, and invasion, upregulate miR-107, and downregulate the levels of related target genes and proteins.

**Conclusion:** Zuojin Pills may exert anti-gastric cancer effects through multiple components, targets, and pathways, and miR-107 is involved in them, which provides theoretical support for its anti-gastric cancer mechanism research and clinical application.

## 003 | Analysis of blood glucose change characteristics during dialysis in patients with maintenance hemodialysis

Li Ma^1^, Dan Liu^1^, Wenhua Liu^2^

^*1*^*Department of Nephrology, Ansteel Group General Hospital, Anshan Liaoning, China,* ^*2*^*Department of Cardiac Surgery, Ansteel Group General Hospital, Anshan Liaoning, China*

**Objective:** The objective is to analyze the characteristics of blood glucose changes during dialysis in patients with maintenance hemodialysis and provide a basis for the clinical prevention and treatment of related complications.

**Methods:** 100 patients who received maintenance hemodialysis in our hospital from January 2022 to December 2023 were selected as the research subjects. All patients used bicarbonate dialysate, and the dialysis time was 3 times a week, 4 hours each time. Before dialysis, 1 hour, 2 hours, 3 hours after the start of dialysis, and at the end of dialysis, venous blood was collected from patients to detect blood glucose levels. At the same time, the patients’ general information, including age, gender, dialysis age, history of diabetes, etc., was recorded, and the correlation between these factors and blood glucose changes during dialysis was analyzed.

**Results:** Before dialysis, the average blood glucose level of the patients was (6.8±2.1) mmol/L. Blood glucose showed a trend of decreasing and increasing during dialysis. After 1 hour of dialysis, blood glucose dropped to the lowest level, with an average of (5.1±1.8) mmol/L, which was significantly different from before dialysis (P<0.05). Subsequently, blood glucose gradually recovered, and the average blood glucose at the end of dialysis was (6.5±2.0) mmol/L, which was significantly different from 1 hour of dialysis (P<0.05). Patients with a history of diabetes had a more significant fluctuation in blood glucose during dialysis (P<0.05), and the longer the dialysis age, the more obvious the blood glucose fluctuation (P<0.05).

**Conclusion:** Blood glucose in patients with maintenance hemodialysis showed a characteristic of first decreasing and then increasing. The history of diabetes and dialysis age were significant factors affecting blood glucose fluctuations. Clinically, the blood glucose monitoring of such patients during dialysis should be strengthened, especially for patients with a history of diabetes and a longer dialysis age, and treatment plans should be adjusted promptly to reduce the adverse effects of blood glucose fluctuations.

## 004 | Analysis of the effect of probiotics-assisted omeprazole-based quadruple regimen on intestinal flora and prognosis in patients with HP-positive gastric ulcer

Mingxing Ma^1#^, Ying Wang^2#^, He Xiao^3^, Xiaoying Wu^4^

^*1*^*Department of Gastroenterology, Xinfa Red Cross Hospital in Daoli District, Harbin, China,* ^*2*^*Liver Disease Center, The Second Affiliated Hospital of Shandong First Medical University, Tai’an, China,* ^*3*^*Department of Encephalopathy, Encephalopathy Center, Tai’an Hospital of Traditional Chinese Medicine, Tai’an, China,* ^*4*^*Department of Emergency, Shanghai Fourth People’s Hospital, School of Medicine, Tongji University, Shanghai, China.* ^*#*^*These authors contribute equally to this study.*

**Objective:** This paper explores the effect of probiotics-assisted omeprazole-based quadruple regimen on intestinal flora and prognosis in patients with Helicobacter pylori (Hp)-positive gastric ulcer.

**Methods:** 120 patients diagnosed with Hp-positive gastric ulcers in our hospital from January 2022 to December 2023 were selected and randomly divided into an observation and a control group, with 60 cases in each group. The control group was treated with a conventional omeprazole-based quadruple regimen, and the observation group was treated with probiotic preparations. The treatment course was 14 days. Before and after treatment, stool samples were collected from patients, and 16S rRNA gene sequencing technology was used to analyze the richness, diversity, and changes in the composition of the intestinal flora. The patients were followed up for 6 months, and the ulcer healing status, Hp eradication rate, recurrence rate, and adverse reactions were recorded.

**Results:** Before treatment, the two groups had no significant differences in the intestinal flora indicators. After treatment, the richness and diversity of intestinal flora in the observation group were significantly improved compared with those in the control group (P<0.05), the relative abundance of beneficial bacteria such as Bifidobacterium and Lactobacillus increased, and the relative abundance of harmful bacteria such as Enterobacter decreased. The ulcer healing rate (90.0%) in the observation group was higher than that in the control group (75.0%), the Hp eradication rate (93.3%) was significantly higher than that in the control group (80.0%), and the recurrence rate (6.7%) was lower than that in the control group (20.0%). The differences were statistically significant (P<0.05). The incidence of adverse reactions in the observation group was lower than in the control group (P<0.05).

**Conclusion:** A probiotic-assisted omeprazole-based quadruple regimen can effectively regulate the intestinal flora of patients with HP-positive gastric ulcers, improve ulcer healing and HP eradication rates, reduce recurrence and adverse reactions, and improve patient prognosis, which has high clinical application value.

## 005 | Evaluation of the effect of 4D-CT scan-based intensity-modulated radiotherapy combined with TACE on the prognosis of patients with rectal cancer liver metastasis

Lu Sun, Hongli Zhao

*Department of Oncology Shandong Provincial Hospital Affiliated to Shandong First Medical University, Jinan, China*

**Objective:** The paper evaluates the effect of 4D-CT scan-based intensity-modulated radiotherapy combined with transarterial chemoembolization (TACE) on the prognosis of patients with rectal cancer liver metastasis.

**Methods:** 80 patients with rectal cancer liver metastasis diagnosed from January 2020 to December 2023 were selected and randomly divided into a combined treatment group and a single-drug TACE group, with 40 cases in each group. The combined treatment group received 4D-CT scan-based intensity-modulated radiotherapy combined with TACE, and the single-drug TACE group only received TACE treatment. The total radiotherapy dose was 50.4Gy, divided into 28 irradiations. TACE uses the Seldinger technique to inject a mixed emulsion of chemotherapy drugs and iodized oil into the hepatic artery. Follow-up after treatment was performed to evaluate tumor changes by enhanced CT or MRI, and progression-free survival (PFS), overall survival (OS), and adverse reactions were recorded. SPSS 23.0 software was used to analyze the data.

**Results:** The median PFS in the combined treatment group was 10.2 months, which was longer than 6.8 months in the single-drug TACE group (P<0.05), the median OS was 20.5 months, which was also significantly longer than 14.3 months in the single-drug TACE group (P<0.05). Regarding adverse reactions, there was no difference in the incidence of common responses such as nausea, vomiting, and fatigue between the two groups (P>0.05). The proportion of radiation-induced liver injury in the combined treatment group was slightly higher, but most of them were mild and could be relieved symptomatically.

**Conclusion:** 4D-CT scanning intensity-modulated radiotherapy combined with TACE can significantly prolong PFS and OS in patients with rectal cancer liver metastasis. Although the risk of radiation-induced liver injury is slightly increased, the overall safety is acceptable, which provides a better treatment plan for improving patient prognosis.

## 006 | Changes of serum interleukin-6, IL-8, IL-10, and TNF-α in children with Kawasaki disease complicated by mycoplasma infection

Xiaolei Wu, Yun Zhao, Lin Cheng, Liang Sun, Caixia Wang, Zixuan Wang

*Department of Emergency Medicine, The Third Hospital of Shandong Province, Ji’nan, China*

**Objective:** This study explored the changes of serum interleukin-6 (IL-6), interleukin-8 (IL-8), interleukin-10 (IL-10), and tumor necrosis factor-α (TNF-α) in children with Kawasaki disease complicated by mycoplasma infection and provided a reference for clinical practice.

**Methods:** A total of 100 children with Kawasaki disease diagnosed from January 2022 to December 2024 were selected and divided into a concurrent infection group (40 cases) and a non-concurrent infection group (60 cases) according to whether they were complicated by mycoplasma infection. Another 50 healthy children were selected as the control group. Enzyme-linked immunosorbent assay (ELISA) was used to detect the levels of IL-6, IL-8, IL-10, and TNF-α in the serum of the three groups of children. SPSS 25.0 software was used for analysis. The measurement data were expressed as mean ± standard deviation. Variance analysis was used for multiple groups, an LSD-t test for pairwise comparison, and Pearson analysis for correlation. P<0.05 was considered statistically significant.

**Results:** The serum levels of IL-6 (56.2±10.5) pg/mL, IL-8 (48.6±8.3) pg/mL, and TNF-α (35.8±7.6) pg/mL) The concurrent infection group was significantly higher than those in the non-concurrent infection group [(38.5±8.1) pg/mL, (30.2±6.5) pg/mL, and (22.4±5.2) pg/mL, respectively]. The non-concurrent infection group was also higher than the control group [(15.3±4.2) pg/mL, (18.7±5.1) pg/mL, and (10.6±3.5) pg/mL, respectively], and the differences were statistically significant (P<0.05). The IL-10 level in the concurrent infection group (28.4±6.8) pg/mL was lower than that in the non-concurrent infection group (35.7±7.2) pg/mL, and the non-concurrent infection group was lower than that in the control group (42.5±8.4) pg/mL, with statistically significant differences (P<0.05). Correlation analysis showed that IL-6, IL-8, and TNF-α were positively correlated with concurrent infection (r=0.68, 0.59, and 0.62, respectively, P<0.05), and IL-10 was negatively correlated with it (r=-0.55, P<0.05).

**Conclusion:** The serum IL-6, IL-8, and TNF-α of children with Kawasaki disease complicated by mycoplasma infection were increased, and IL-10 was decreased.

## 007 | Application of neoadjuvant immunotherapy in locally advanced head and neck cancer: Optimization of the balance between surgery and quality of life

Xu Zhang^#^, Qian Zhang^#^, Juan Shen, Hongmei Wang

*Department of General Medicine, The 8th Medical Center of PLA General Hospital, Beijing, China.* ^*#*^*These authors contribute equally to this study.*

**Objective:** This article explores the effect of neoadjuvant immunotherapy on surgery and quality of life of patients with locally advanced head and neck cancer and optimizes the balance between the two.

**Methods:** 80 patients with locally advanced head and neck cancer from 2020 to 2023 were selected and randomly divided into a combined and a control group, 40 cases in each group. The combined group received three cycles of pembrolizumab immunotherapy before surgery, once every 3 weeks. The two groups’ surgical resection rates, durations, and bleeding volumes were evaluated. The patients were followed up for 1 year after surgery, and the EORTC QLQ-C30 and EORTC QLQ-H&N35 scales assessed the quality of life. The progression-free survival (PFS) and overall survival (OS) were recorded. SPSS 24.0 software was used for analysis, and the measurement data were expressed as mean ± standard deviation, and the independent sample t test was performed, the counting data were expressed as a rate (%), and the χ² test was used, and P<0.05 was considered statistically significant.

**Results:** The surgical resection rate of the combined group was 90.0%, higher than 70.0% in the control group (P<0.05). The operation time of the combined group [(180.5±25.6)min] was shorter than that of the control group [(210.3±30.2)min], and the intraoperative blood loss [(250.8±40.5)mL] was less than that of the control group [(320.6±50.3)mL], with statistically significant differences (P<0.05). During the follow-up period, the scale score of the combined group was better than that of the control group (P<0.05), and the median PFS (12.5 months) and median OS (20.8 months) were longer than those of the control group (8.2 months and 15.6 months), with statistically significant differences (P<0.05).

**Conclusion:** Neoadjuvant immunotherapy can improve the surgical resection rate of local advanced head and neck cancer, shorten the operation time, reduce bleeding, improve the quality of life, prolong PFS and OS, and provide patients with a better treatment plan.

## 008 | Analysis of risk factors and predictive value of minimally invasive treatment of ovarian torsion necrosis with pethidine hydrochloride combined with laparoscopic surgery

Weihang Zhang^1^, Lihua Sun^2^, Ranran Yue^3^, Guoqing Cao^4^, Xuehong Li^5^

^*1*^*Department of Gynaecology, Affiliated Jinhua Hospital, Zhejiang University School of Medicine, Jinhua, China,* ^*2*^*Department of Medical Imaging Technology Binzhou Polytechnic, BinZhou, China,* ^*3*^*Pharmacy Teaching and Research Department, Binzhou Polytechnic, Binzho, China*

^4^Department of Medical and Dental Prosthetic Technology, Binzhou Polytechnic, Binzhou, China, ^5^Gynecology LinHai Hospital of traditional Chinese medicine, Linhai China

**Objective:** This paper explores the risk factors and predictive value of pethidine hydrochloride combined with laparoscopic surgery to treat ovarian torsion necrosis and assist clinical decision-making.

**Methods:** The data of 120 patients with ovarian torsion who received the above treatment in our hospital from January 2018 to December 2023 were reviewed. Intraoperative pathology divided them into the ovarian torsion necrosis group (30 cases) and the non-necrosis group (90 cases). Information such as patient age, duration from onset to visit, serum CA125 level, ovarian blood flow under ultrasound, and degree of torsion were collected. The associated factors were first screened by univariate analysis. Then, the independent risk factors were determined by multivariate logistic regression, and the receiver operating characteristic (ROC) curve was used to evaluate its predictive value.

**Results:** Univariate analysis showed that the proportion of ≥24 hours from onset to visit, serum CA125≥100 U/mL, disappearance of ovarian blood flow signal, and torsion ≥360° in the necrosis group was higher than that in the non-necrosis group (P<0.05). Multivariate Logistic regression showed that ≥24 hours from onset to visit (OR = 3.56, 95% CI: 1.89 - 6.71) and disappearance of ovarian blood flow signal (OR = 4.28, 95% CI: 2.15 - 8.53) were independent risk factors. The ROC curve showed that the area under the curve of the time from onset to visit combined with ovarian blood flow signal to predict ovarian torsion necrosis was 0.856 (95% CI: 0.782 - 0.930), with good predictive value.

**Conclusion:** ≥24 hours from onset to visit and disappearance of ovarian blood flow signal are independent risk factors for ovarian torsion necrosis. Combining the two can better predict necrosis, which is conducive to early clinical detection and intervention.

## 009 | Analysis of the efficacy of radiotherapy combined with ametinib in the treatment of stage III EGFR sensitive mutation lung cancer

Gang Liu^1^, Jingyi Wang^2^, Fengchao Wang^3^, Meng Xu^4^

^*1*^*Department of Oncology Laibin People’s Hospital, Laibin, China,* ^*2*^*Department of Radiation Oncology,Harbin Medical University Cancer Hospital, Harbin, China,* ^*3*^*Ward 6-Shanghai Goldberg Cancer Hospital, Shanghai, China,* ^*4*^*Clinical pharmacology-Affiliated Hospital of Qinghai University, Xining, China*

**Objective:** This article explores the efficacy of radiotherapy combined with imatinib in stage III EGFR-sensitive mutation lung cancer and provides new ideas for clinical practice.

**Methods:** 80 patients with stage III EGFR-sensitive mutation lung cancer diagnosed from 2020 to 2023 were selected and randomly divided into a combination group and a single-drug group, with 40 cases in each group. The single-drug group took ametinib orally, 110 mg/day, the combination group received radiotherapy on this basis, with a total dose of 60 Gy, divided into 30 irradiations. Chest CT examinations were performed regularly during treatment, and the efficacy was evaluated according to the RECIST version 1.1, which was divided into complete remission (CR), partial remission (PR), stable disease (SD), and progressive disease (PD). At the same time, adverse reactions such as rash, diarrhea, and radiation pneumonia were recorded. SPSS 22.0 software was used for analysis, and the efficacy and incidence of adverse reactions between the groups were tested by χ² test. P<0.05 was considered statistically significant.

**Results:** The objective response rate (ORR, CR+PR) of the combined group was 75.0%, which was higher than 50.0% of the single-drug group (P<0.05), the disease control rate (DCR, CR+PR+SD) was 90.0%, which was also higher than 70.0% of the single-drug group (P<0.05). Regarding adverse reactions, the incidence of rash and diarrhea in the two groups was similar (P>0.05), and the incidence of radiation pneumonitis in the combined group was 15.0%, higher than 5.0% of the single-drug group. Still, most of them were mild and could be relieved symptomatically.

**Conclusion:** Radiotherapy combined with imatinib can significantly improve the objective response rate and disease control rate in the treatment of stage III EGFR-sensitive mutation lung cancer. Although the incidence of radiation pneumonitis increased, it was overall safe and controllable, providing a better treatment plan for this type of patient.

**Acknowledgements:** This work was supported by the Guangxi Zhuang Autonomous Region Health Commission self-funded scientific research projects (Z-G20221781).

## 010 | Clinical observation of Xinshangxuduan Decoction combined with operation in the treatment of Colles fracture based on network pharmacology

Huadong Lu, Gengqi Wang

*Orthopedic and traumatology, Yueyang integrated traditional Chinese and Westen Medicine Hospital affiliated to Shanghai University of traditional Chinese Medicine, Shanghai, China*

**Objective:** To observe the clinical efficacy of Xinshang Xuduan decoction combined with surgical treatment for Colles fracture based on network pharmacology.

**Methods:** This study enrolled 94 patients with Colles fractures admitted to the Orthopedics Department of our hospital from December 2021 to November 2023 and randomly divided them into two groups. The control group received manual closed reduction combined with external fixation after surgery, combined with conventional anti-inflammatory and pain relief, and early functional exercise treatment, The experimental group received oral administration of Xinshang Xuduan Tang in addition to conventional treatment after the combination external fixation stent surgery. By comparing the postoperative pain level, swelling level, clinical healing time of fractures, relevant imaging indicators, and wrist joint function scores between the two groups.

**Results:** After 8 weeks of treatment, Mann Whitney U test was performed on the wrist joint function score data of both groups of patients, Z=-2.479, P=0.013<0.05, There is statistical significance, and the excellent rate of the experimental group is significantly higher than that of the control group, indicating that the experimental group is more effective in wrist joint function recovery. After 12 weeks of treatment, Mann Whitney U test was performed on the wrist joint function score data of both groups of patients, Z=-2.029, P=0.042<0.05, There is statistical significance, and the excellent rate of the experimental group is higher than that of the control group. The experimental group has a more excellent therapeutic effect in the long-term recovery of wrist joint function. The clinical healing time data of two groups of patients with fractures were processed, and both groups of data followed a normal distribution with equal variance. The t-test results showed P<0.05, indicating a significant difference, indicating that the herbal formula used in the experimental group has a promoting effect on fracture healing.

**Conclusion:** Traditional Chinese Medicine has a unique theoretical system in the treatment of traumatic fractures, which has gradually developed and formed in the process of combating traumatic diseases. It is widely used in clinical practice and has excellent therapeutic effects. Modern traditional Chinese medicine has summarized the effective treatment experience of predecessors and proposed the three-stage syndrome differentiation theory for fracture treatment, which plays an important role in the treatment of traumatic fractures.

## 011 | Dent disease misdiagnosed with purpura nephritis: a case report and analysis

Zhe Liu, Wenhong Wang

*Department of Nephrology Tianjin Children’s Hospital (Children’s Hospital, Tianjin University) Tianjin Key Laboratory of Birth Defects for Prevention and Treatment, Tianjin, China*

**Objective:** This article reports a case of Dent’s disease misdiagnosed as purpura nephritis and conducts literature analysis.

**Methods:** The patient is an 11-year-old male who has been receiving outpatient treatment at a local hospital for over 5 months due to rash on both lower limbs and over 2 months due to proteinuria. The rash presents as a symmetrical hemorrhagic rash on both lower limbs, gradually spreading to the buttocks and upper limbs. There is no joint swelling, limited mobility, abdominal pain, vomiting, or gastrointestinal bleeding, no oral ulcers, no scrotal swelling, no edema, abnormal urine color, or reduced urine volume.

**Results:** Based on comprehensive clinical manifestations, auxiliary examination features, pathological characteristics, and gene sequencing results, the final diagnosis is Dent disease type 1. Afterwards, stop using mycophenolate mofetil and gradually reduce hormone intake. At the same time, take hydrochlorothiazide orally to reduce urinary calcium excretion and prevent kidney stone formation with potassium citrate. At present, there is no obvious hematuria in the outpatient follow-up. The 24-hour urine protein is 1197-2152mg, the 24-hour urine calcium is 7.2-1.1mg/kg, the serum electrolytes are normal, and the creatinine is 61-64 μ mol/L, eGFR 85~89ml/(min·1.73m^2^).

**Conclusion:** For any level of proteinuria, urine protein electrophoresis and LMWP testing should be important diagnostic references. Massive proteinuria and hematuria with proteinuria should not be used as a basis for excluding renal tubular disease. At the same time, the diagnosis of IgAVN is based on pathological changes. For moderate to high levels of proteinuria and even renal proteinuria, especially when deciding to use hormone and/or immunosuppressive therapy, timely kidney biopsy should be performed to compensate for the shortcomings of clinical diagnosis and avoid additional harm caused by drugs.

**Acknowledgements:** This work was supported by the Tianjin Key Medical Discipline (Specialty) Construction Project (TJYXZDXK-040A).

## 012 | Correlation analysis of rheumatoid arthritis disease progression with serum chemokines, RNAKL, 25-hydroxyvitamin D levels and miR223_5p

Ming Zhang^12^, Haiqin Zhu^2^, Shuhong Zhou^3^

^*1*^*The First Clinical Medical College of Gansu University of Chinese Medicine, Lanzhou, China,* ^*2*^*Department of Rheumatology and immunology, Zhangye Second People’s Hospital, Zhangye, China,* ^*3*^*Department of Immunorheumatology, Gansu Provincial Hospital, Lanzhou, China*

**Objective:** To investigate the correlation between serum chemokines, miRNA223-5p, and 25 hydroxyvitamin D (25 hydroxyvitamin D, 25 (OH) D) levels and the progression of rheumatoid arthritis.

**Methods:** 289 patients with rheumatoid arthritis treated in our hospital from January 2022 to December 2023 and 100 healthy individuals who underwent physical examinations in our hospital during the same period were selected for the study. Using 28 joint disease activity scores, RA patients were divided into RA remission group and RA activity group. Monitor fasting serum levels of C-C chemokine ligand 20 (CCL20), miRNA223-5p, and 25 (OH) D in RA patients and healthy individuals, and analyze the correlation between these indicators and the progression of RA disease.

**Results:** Compared with the control group of healthy subjects, the serum levels of CCL20 and miRNA223-5p in RA patients were significantly increased, while the level of 25 (OH) D was significantly decreased, and the differences were statistically significant (P<0.05), The serum levels of CCL20 and miRNA223-5p in the RA active group were significantly higher than those in the RA remission group, and the serum levels of 25 (OH) D were significantly lower in the RA active group than in the RA remission group, with statistically significant differences (P<0.05), Correlation analysis showed that serum CCL20 and miRNA223-5p levels were positively correlated with RA disease progression (P<0.05), while serum 25 (OH) D levels were negatively correlated with RA disease progression (P<0.05).

**Conclusion:** Serum levels of CCL20 and miRNA223-5p are positively correlated with the progression of RA disease, while serum levels of 25 (OH) D are negatively correlated with RA disease progression. CCL20, miRNA223-5p, and 25 (OH) D can serve as biological indicators for monitoring disease activity, assisting doctors in comprehensively and comprehensively assessing the patient’s condition.

## 013 | Effect of montmorillonite powder combined with norfloxacin on intestinal flora imbalance and Th17Treg cell immune balance in the treatment of enteritis

Zhuoxun Liu^1^, LiHuaSun^2^, Ran ran Yue^3^, Guoqing Cao^4^, WeiBin Guo^5^

^*1*^*Department of Gastroenterology, People’s hospital in Hegang city, Hegang, China,* ^*2*^*Department of Medical Imaging Technology-Binzhou Polytechnic, BinZhou, China,* ^*3*^*Pharmacy Teaching and Research Department, Binzhou Polytechnic, Binzhou, China,* ^*4*^*Department of Medical and Dental Prosthetic Technology, Binzhou Polytechnic, Binzhou, China,* ^*5*^*Department of Health, Binzhou Center for Disease Control and Prevention, BinZhou, China*

**Objective:** This study explores the effect of montmorillonite powder combined with norfloxacin in treating enteritis on intestinal flora and Th17/Treg cell immune balance.

**Methods:** 120 patients with enteritis diagnosed from 2022 to 2024 were selected and randomly divided into a combined group, a montmorillonite powder monotherapy group, and a norfloxacin monotherapy group, with 40 cases in each group. The combined group was treated with montmorillonite powder (3g/time, 3 times/day) and norfloxacin (0.3g/time, 2 times/day), the monotherapy group used the corresponding drugs alone, and the course of treatment was 7 days. Before and after treatment, stool samples were taken from patients, and intestinal flora was analyzed by 16S rRNA gene sequencing, peripheral blood was collected, and the proportion of Th17 and Treg cells was measured by flow cytometry, and the Th17/Treg ratio was calculated to evaluate immune balance. SPSS 23.0 software was used for analysis, and variance analysis and LSD-t test were used between groups. P<0.05 was considered statistically significant.

**Results:** There were no significant differences in the relevant indicators among the three groups before treatment. After treatment, the richness and diversity of intestinal flora in the combined group were better than those in the single-drug group (P<0.05), the abundance of beneficial bacteria such as Bifidobacterium and Lactobacillus increased, and the abundance of harmful bacteria such as Escherichia coli decreased. The proportion of Th17 cells in the combined group decreased, the proportion of Treg cells increased, and the Th17/Treg ratio tended to be normal, statistically significant compared with the single-drug group (P<0.05).

**Conclusion:** Montmorillonite powder combined with norfloxacin is superior to single drugs in regulating intestinal flora and balancing Th17/Treg cell immunity in the treatment of enteritis, which can more effectively improve the patient’s condition and provide a better solution for clinical treatment.

## 014 | Baseline Characteristics and Clinical Implications of Different Stages of Cardiovascular-Kidney-Metabolic Syndrome: A Nationwide Cross-sectional Study

Zhihang Du^1^, Xiaolin Wang^1^, Xueyu Lv^1^, Xu Liu^2^, Dan Li^1^

^*1*^*Department of Cardiology, The Affiliated Hospital of Qingdao University, Qingdao, China,* ^*2*^*Department of Cardiovascular Surgery, The Affiliated Hospital of Qingdao University, Qingdao, China*

**Objective:** This study aimed to evaluate differences in demographic, metabolic, and psychological characteristics between patients with advanced stage (stages 3–4) and early stage (stages 0–2) cardiovascular-kidney-metabolic (CKM) syndrome from an integrated management perspective. By analyzing baseline traits, we sought to provide targeted insights for prevention and intervention strategies for CKM syndrome.

**Methods:** A total of 1,320 individuals participating in the National Health and Nutrition Examination Survey (NHANES) were divided into two distinct groups based on the staging criteria established by the American Heart Association (AHA). Specifically, 509 participants were classified as belonging to the advanced CKM group, while the remaining 811 were assigned to the early CKM group.Data on demographics (age, sex, race), metabolic parameters (BMI, blood glucose, blood pressure, lipid profile), renal function (estimated glomerular filtration rate [eGFR], CKD risk stratification), and depressive symptoms (assessed via PHQ-9) were collected. Descriptive statistics for baseline characteristics were reported as mean (standard error) for continuous variables and unweighted frequencies (weighted percentages) for categorical variables. To account for the complex sampling design of NHANES, all analyses incorporated survey weights to generate nationally representative estimates. Weighted t-tests or weighted chi-square tests were used to compare differences in key variables across groups (e.g., sex, race/ethnicity). For continuous variables with non-normal distributions, the weighted Wilcoxon rank-sum test was employed.

**Results:** Compared with the early CKM group, participants in the advanced CKM group exhibited significantly higher age, male proportion, BMI, triglycerides, waist circumference, fasting glucose, prevalence of hypertension, diabetes, cardiovascular disease, chronic kidney disease, smoking rates, and low-income status. Conversely, eGFR, HDL-C, and total cholesterol levels were lower. Additionally, the advanced CKM group had a markedly higher incidence of clinically significant depressive symptoms (PHQ-9 ≥10, p< 0.01). Significant differences were observed across demographic, metabolic, and lifestyle factors between groups.

**Conclusion:** Our findings reveal that advanced CKM patients present with more adverse metabolic profiles, lower socioeconomic status, and higher depressive risk. These rsults underscore the critical need for multidimensional early identification and intervention. Tailored public health policies and stage-specific clinical management strategies are essential to mitigate the burden of CKM syndrome on individuals and healthcare systems.

## 015 | Turning Point of Complement C1q: A Novel Biomarker for Coronary Collateral Circulation Injury

Xueyu Lv, Yingying Zhang, Zhihang Du, Dan Li

*Department of Cardiology, The Affiliated Hospital of Qingdao University, Qingdao, Shandong, China*

**Objective:** Building on emerging evidence of inflammatory-coronary artery crosstalk, we sought to characterize the relationship between C1q, as an initiating factor of the complement cascade, and coronary collateral circulation (CCC) in chronic total occlusion patients.

**Methods:** In this cross-sectional study (Qingdao, 2024-2025), we enrolled 293 patients with at least 1 chronic total occlusion on coronary angiography. CCC adequacy was dichotomized via Rentrop grading (0-1=poor, 2-3=good). Serum C1q quantification employed the Abbott ARCHITECT® chemiluminescent platform. Analytical innovations included: (1) Threshold detection using adaptive spline modeling (RCS with 3 knots), (2) Predictive validation through bootstrapping (1000 iterations).

**Results:** When study participants were grouped based on the formation of collateral circulation, C1q was significantly higher in the poor CCC formation group compared with the good CCC group (P<0.001).Multifactorial logistic regression analysis was performed using good or poor formation of collateral circulation as the dependent variable, and the results of this analysis showed that C1q (OR 0.98, 95% CI 0.98-0.99, P<0.001) was independent correlate affecting the formation of CCC. Our study revealed a U-shaped association between serum C1q and poor coronary collateral circulation (P<0.001). Below 150 mg/L, increasing C1q (100-150 mg/L) correlated with higher collateral insufficiency risk (β=0.17, P=0.003). Beyond 150 mg/L, the risk trend reversed (Δβ=0.24, P=0.011), indicating uncertainty in high-concentration effects. C1q ≥167.5 mg/L predicted collateral impairment with 71.1% sensitivity and 67.1% specificity (adjusted OR=1.02 per mg/L, P<0.001). A combined model integrating hemodynamic and neuroendocrine factors outperformed C1q alone (AUC=0.80 vs. 0.70, P<0.001), supporting multimodal risk stratification in chronic total occlusion.

**Conclusion:** Our findings unveiled a C1q threshold phenomenon, where 167.5 mg/L emerges as a tipping point for collateral impairment. We propose a monitoring strategy based on complement C1q: For G1/ G2, be vigilant regarding the tracking of C1q (with levels ≥180 mg/ L), and for G3, conduct a multimodal evaluation that incorporates C3a and TNF - α. The next phase of research could employ sample size expansion or stratified analyses based on inflammatory factors and renal function to further elucidate the association between complement C1q and coronary collateral circulation (CCC) formation.

## 016 | Effect of recombinant human brain natriuretic peptide combined with sacubatrotrivalsartan on ventricular remodeling in emergency patients with heart failure

Qing He^1^, Yijiang Du^2^, Yajuan Chen^1^, Ying Meng^1^, Xiaoyan Zhang^1^, Xiang Guo^3^, Zhenyan Fu^1^

^*1*^*Department of Emergency, Wuhan Central Hospital, China Construction Third Engineering Division, Wuhan, China,* ^*2*^*Department of Surgery, Wuhan Central Hospital, China Construction Third Engineering Division, Wuhan, China,* ^*3*^*Department of Emergency, Changsha Taihe Hospital, Changsha, China*

**Objective:** To explore the therapeutic effect of recombinant human brain natriuretic peptide combined with sacubitril/valsartan in emergency patients with heart failure and its influence on ventricular remodeling.

**Method:** A total of 86 patients with emergency heart failure from June 2023 to August 2024 were selected as the subjects and divided into two groups, with 43 cases in each group, by the envelope method. Both groups were treated with conventional methods. The control group was intervened with recombinant human brain natriuretic peptide, while the observation group was intervened with sacubitril/valsartan. The effects of the patients in both groups were evaluated after 4 weeks of treatment. The ventricular remodeling, biochemical indicators and the incidence of adverse reactions were compared between the two groups.

**Result:** Ventricular remodeling was alleviated after intervention in both groups. The IVST and LVPWT in the observation group were lower than those in the control group (P<0.05). LVMI was higher than that of the control group (P<0.05), The biochemical indicators were reduced after the intervention of the two groups. The levels of NT-proBNP, MMP-9, IL-6 and RBP4 in the observation group were lower than those in the control group (P<0.05). There was no statistical difference in the safety of the two groups of drugs (P>0.05).

**Conclusion:** Recombinant human brain natriuretic peptide combined with sacubitril/valsartan can alleviate ventricular remodeling in patients with emergency heart failure, improve the biochemical index levels of patients, and has high drug safety. It is worthy of promotion and application.

## 017 | Study on the improvement of pelvic floor electromyography in functional anorectal pain through combined treatment with pinus tabuliformis extract and pelvic floor biofeedback

Lei Tong^1^, Mengting Qin^1^, Ruyun Cai^1^, Zhonghua Hong^1^, Qi Wang^1^, Zhenya Zhu^2^

^*1*^*Department of Anorecta, Jiaxing Hospital of Traditional Chinese Medicine, Jiaxing, China,* ^*2*^*Orthopedics and Traumatology, Jiaxing Hospital of Traditional Chinese Medicine, Jiaxing, China*

**Objective:** Functional anorectal pain (FARP) is a common yet frequently misdiagnosed condition, primarily associated with pelvic floor muscular dysfunction. This study aimed to evaluate the efficacy of a treatment combining Pinus tabuliformis extract with pelvic floor biofeedback in improving electromyographic activity of the pelvic floor in patients with FARP.

**Methods:** In this prospective, randomized controlled trial, 36 patients diagnosed with FARP at our hospital’s colorectal outpatient clinic were enrolled. They were randomly assigned to either the treatment group, which received Pinus tabuliformis extract combined with pelvic floor biofeedback, or the control group, which received conventional therapy alone. Both groups were evaluated using the Visual Analogue Scale (VAS) and surface electromyography (sEMG) of the pelvic floor muscles before and after the treatment. Data were analyzed using the SPSS statistical software package.

**Results:** Both groups demonstrated significant improvements in VAS scores post-treatment, indicating effective pain relief. The treatment group showed a more substantial reduction in pain severity, with VAS scores decreasing significantly (P<0.001), compared to the control group (P<0.05). sEMG assessments revealed significant enhancements in pelvic floor muscle activity, with the treatment group displaying greater improvements in Resting AVG, AVG Peak, and Intermittent AVG. Specifically, the treatment group’s Resting AVG decreased from 10.28 μV to 8.56 μV (P<0.001), AVG Peak increased from 61.34 μV to 74.10 μV (P<0.001), and Intermittent AVG from 47.80 μV to 55.21 μV (P<0.001). The control group also showed improvements but to a lesser extent. The Coefficient of Variability (CV) also changed more significantly in the treatment group, indicating increased consistency and stability in muscle contractions.

**Conclusion:** The study confirms that combining Pinus tabuliformis extract with pelvic floor biofeedback therapy significantly enhances the treatment effects in patients with FARP compared to biofeedback therapy alone. These findings suggest that the addition of Pinus tabuliformis extract can further improve pelvic floor muscle function and pain relief, demonstrating its potential as an effective component in treating FARP.

**Acknowledgements:** This study was supported by the Zhejiang Province Traditional Chinese Medicine Science and Technology Plan Projects (No.2023ZL697).

## 018 | The Efficacy of Qi-Invigoration, Yin-Nourishing, and Blood-Dispelling Chinese Herbal Compound in Treating Ventricular Premature Beats: A Meta-Analysis

Xinrui Cui^1^, Sijia Liang^1^, Lihong Gong^2^

^*1*^*Liaoning University of Traditional Chinese Medicine, Shenyang, China,* ^*2*^*Department of Cardiology, the Affiliated Hospital of Liaoning University of Traditional Chinese Medicine, Shenyang, China*

**Objective:** Ventricular Premature Beats (VPB) are a common arrhythmia characterized by premature ventricular contractions occurring on the basis of a normal rhythm. The incidence of VPB is relatively high and may lead to symptoms such as palpitations and arrhythmias, with severe cases potentially increasing the risk of heart failure and sudden cardiac events. With the accelerating pace of modern life and increased stress, the incidence of VPB is on the rise, making it an important issue in the management of clinical cardiovascular diseases. Preliminary evidence suggests that Chinese herbal medicine has the potential to enhance therapeutic efficacy and reduce the incidence of adverse reactions. The aim of this study is to systematically evaluate the efficacy of the Chinese herbal compound of invigorating Qi, nourishing Yin, and dispelling blood stasis in the treatment of VPB through a meta-analysis, to provide scientific evidence for its clinical application, and to lay a foundation for the promotion of the integrated treatment model of Chinese and Western medicine.

**Methods:** A total of 3021 articles were initially retrieved and deduplicated using EndNote X9 reference management software, leaving 2562 articles. A preliminary screening process, involving the reading of titles, abstracts, and keywords, excluded studies unrelated to the research topic, leaving 415 articles for further review. After conducting full-text downloads and detailed assessments, a total of 44 eligible studies were ultimately included in the meta-analysis. Data analysis was conducted using RevMan 5.4 software. Primary statistical measures included overall efficacy and VPB efficacy, which were summarized using risk ratios (RR) and 95% confidence intervals (CIs). Heterogeneity was assessed using the I² statistic and the Q test. If I²<50% and P>0.10, indicating low heterogeneity, a fixed-effect model was used. If I² ≥ 50% or P ≤ 0.10, indicating significant heterogeneity, a random-effects model was used, and sources of heterogeneity were further explored.

**Results:** The forest plot shows that “Qi-invigorating, Yin-nourishing, and Blood-dispelling Chinese herbal compound” significantly outperforms the control group in improving the electrocardiographic efficacy rate for VPB treatment (OR=3.36, 95% CI [2.72, 4.16], P<0.0001). Heterogeneity analysis revealed an I² of 0% and a P-value of 0.69, indicating no significant heterogeneity among the included studies, with highly consistent results, thus supporting the use of a fixed-effect model. The effect sizes reported in most studies were close to the pooled effect size, and the 95% CI of all studies did not cross 1, indicating that the treatment has a significant effect in improving the electrocardiographic efficacy rate, with reliable and robust results.

**Conclusion:** In conclusion, Qi-invigorating, Yin-nourishing, and Blood-dispelling Chinese herbal medicine demonstrates significant therapeutic advantages in the treatment of ventricular premature beats (VPB). Despite some limitations in the included studies, this meta-analysis provides important evidence supporting the role of traditional Chinese medicine in the treatment of arrhythmias. Future research should adopt more rigorous and systematic approaches to further clarify its clinical value and underlying mechanisms of action, thereby promoting the development of integrative medicine.

## 019 | Applications of Metabolomics in Atrial Fibrillation Research

Sijia Liang^1^, Xinrui Cui^1^, Lihong Gong^2^

^*1*^*Liaoning University of Traditional Chinese Medicine, Shenyang, China,* ^*2*^*The Affiliated Hospital of Liaoning University of Traditional Chinese Medicine, Shenyang, China*

**Objective:** Atrial fibrillation (AF) is the most common supraventricular tachyarrhythmia, characterized by a rapid and irregular atrial rhythm. During AF, homeostasis is disrupted, and changes in metabolites can further elucidate pathogenic mechanisms, pharmacological mechanisms, and assist in early diagnosis. The holistic, integrative, and dynamic nature of metabolomics aligns highly with the TCM philosophy of “syndrome differentiation and holistic treatment.” Scholars in TCM have applied this technology to the research of TCM diagnosis and treatment of AF.This review summarizes recent studies on myocardial metabolism and the application of metabolomics in AF, aiming to provide references for the clinical diagnosis and treatment of AF.

**Methods:** In recent years, an increasing body of evidence has highlighted the close association between AF and metabolic disorders. The onset and progression of AF involve the collective perturbation of metabolites across multiple metabolic pathways. Metabolomics, with its holistic perspective, provides powerful tools for multi-faceted and comprehensive research on AF. It not only provides a direct visualization of the metabolic state of the body and reflects dynamic pathological and physiological changes through metabolic pathways but also enables integrative multi-omics analysis.

**Results:** In summary, AF patients exhibit abnormalities in fatty acid metabolism, amino acid metabolism, and bile acid metabolism. The metabolic profiles of different TCM syndromes show significant differences, and DMs have the potential to become biomarkers for diagnosing these syndromes.

**Conclusion:** First, although key metabolites have been identified through differential analysis and potential biomarkers have been pinpointed, significant discrepancies exist across studies. Large-scale research is needed to validate the feasibility of these findings for early AF diagnosis. Second, most current studies have small sample sizes and diverse sample types, leading to considerable variations in identified DMs. Distinguishing, correcting, and integrating these different results remains a significant challenge. Third, given the complex pathogenesis of AF, inferring pathological mechanisms solely from key metabolites is extremely difficult. Therefore, integrating metabolomics with other omics approaches, such as transcriptomics, genomics, and proteomics, is essential to better elucidate the pathogenesis of AF and provide references for clinical diagnosis and treatment.

## 020 | Meta-analysis of the effect of Ureaplasma urealyticum infection on male semen quality

Lei Han

*Department of Disinfection, Beijing Haidian District Center for Disease Control and Prevention, Beijing, China*

**Objective & Methods:** To systematically review the effect of Ureaplasma urealyticum infection on male semen quality of The Chinese and English literatures on the effect of Ureaplasma urealyticum infection on male semen quality were searched in the databases of Science (WOS), Cochrane Library, China National Knowledge Infrastructure (CNKI), Wanfang, etc. The search time was from the establishment of the database to January 2025. The RevMan5.3 software was used to perform Meta-analysis on each study.

**Results:** A total of 2617 literatures were initially obtained, 1846 literatures were screened, 40 literatures were obtained after rescreening, and 12 literatures were finally included, 8 literatures involved the effect of Ureaplasma urealyticum infection on male semen quality, and the results showed that Ureaplasma urealyticum infection can cause male semen quality to decline, 7 literatures involved the effect of Ureaplasma urealyticum infection on male infertility, and the results showed that Ureaplasma urealyticum infection can increase the incidence of male infertility.

**Conclusion:** Ureaplasma urealyticum infection can affect the quality of male semen, cause sperm head deformity, mainly acrosome region deletion and microcephaly, resulting in decreased semen quality, which is one of the important causes of male infertility. However, given the quantity and quality of the included literature, further research and discussion is needed.

## 021 | Comparison of the efficacy of laparoscopic lateral suspension of the abdominal wall of the uterus and uterosacral fixation in the treatment of pelvic floor organ prolapse

Haiying Wang, Xiaomei Peng

*Chengdu Fifth People’s Hospital, Chengdu, China*

**Objective:** To compare and analyze the therapeutic effects of laparoscopic transverse suspension of the abdominal wall and uterosacral cape suspension in the treatment of pelvic organ prolapse.

**Methods:** According to the random number method, 80 patients with uterine prolapse in our hospital from November 2018 to January 2023 were divided into two groups: the first group was the control group, and the second group was the observation group, with 40 cases in each group. The observation group received laparoscopic uterine abdominal wall suspension surgery for treatment, while the control group received uterine sacral cape suspension surgery to complete the treatment plan.

**Results:** Compare the treatment effects of two groups of patients with pelvic floor organ prolapse. The total hospitalization time of the patients was observed throughout the entire process, and it was found that the surgical time of the control group was longer than that of the observation group, and the amount of bleeding was also greater than that of the observation group, with statistical significance (P<0.05). Based on different surgical treatments applied to the control group and observation group, relevant measures can be taken to identify the current situation and adverse problems of the patients. Currently, no complications have been found.

**Conclusion:** Laparoscopic uterine abdominal wall suspension surgery can reduce surgical time and hospitalization time for patients, as well as significantly reduce surgical bleeding. This can reduce the damage rate of surgery to patients, lower medical expenses, and avoid complications related to the internet, promoting better quality of life and treatment outcomes for patients.

## 022 | The Association of Carotid Atherosclerotic Plaques and Their Stability with Cognitive Dysfunction after Acute Ischemic Stroke

Yong Wu, Rong Xiang,Wei Fu,Zhuo Chen, Qi Zhou

*China Sichuan Mianzhu people* ^,^ *s Hospital, Mianzhu, China*

**Objective:** To explore the correlation of carotid stiffness plaque and cognitive dysfunction after acute ischemic stroke.

**Methods:** For 80 patients with carotid sclerosis plaque treated in hospital from January 2023 to January 2024, 40 patients with acute ischemic stroke without carotid sclerosis were selected in the ratio of 2:1. All patients received the Montreal cognitive assessment scale (MoCA) to evaluate their cognitive function, and the association between carotid sclerosis plaque stability, stenosis degree and lesion site and the MoCA were analyzed.

**Results:** MOCA evaluates the cognitive function, The MOCA score and score of the study group were lower than the control group (*P*<0.05), The total score and each dimension score of the patients in the stable plaque group were higher than that of the vulnerable plaque group (*P*<0.05), The MOCA total score, spatial executive ability, attention, orientation force, delayed memory score were higher than moderate stenosis and severe stenosis, moderate stenosis than severe stenosis (*P*<0.05), and naming, abstract thinking, verbal fluency score were higher than moderate and moderate stenosis (*P*<0.05), but there was no difference between patients with moderate stenosis and severe stenosis (*P*>0.05). In the right stenosis group, patients had higher MOCA scores, spatial executive ability, verbal fluency, and delayed memory scores than in the left and bilateral stenosis groups, And the left stenosis group was higher than the bilateral stenosis group (*P*<0.05), The right stenosis group had higher attention and abstract thinking scores than the left and bilateral stenosis group (*P*<0.05), However, there was no difference between the left stenosis and bilateral stenosis groups (*P*>0.05), There was no difference in directional force score between the right and left stenosis groups (*P*>0.05), However, all were higher than the bilateral stenosis group (*P*<0.05), No difference was found between the three groups (*P*>0.05),After the correlation analysis, the total MOCA score of acute ischemic stroke patients showed a high correlation with the carotid sclerosis plaque stability, degree of stenosis, and site of the lesion.

**Conclusion:** carotid clerotic plaque and its stability and degree of stenosis have significant effect on cognitive dysfunction after acute ischemic stroke.

## 023 | Observation on the efficacy of Wassel classification-assisted replantation of multiple finger amputations in the treatment of congenital thumb deformities in children

Zhiqiang Fan

*Yibin Second People’s Hospital, Sichuan, Yibin, China*

**Objective:** To explore the efficacy of Wassel classification-assisted polydactyly replantation in the treatment of congenital thumb deformity in children.

**Methods:** A total of 64 children with congenital thumb deformity admitted to our hospital from November 2019 to December 2021 were selected as the subjects of this study, and all children underwent Wassel classification-assisted polydactyly replantation. The hospitalization time and wound healing time of children with different Wassel classifications were compared, and the thumb function (modified Tada score) of children with different Wassel classifications after treatment was evaluated. The finger activity and finger grip strength levels of children before and after treatment were compared, and the prognosis and recovery of children were evaluated.

**Results:** There was no significant difference in the hospitalization time and wound healing time of children with different Wassel classifications (*P*>0.05). after surgical treatment, 40 cases were excellent, 22 cases were good, and 2 cases were poor, with an excellent and good rate of thumb function of 96.87%. the finger mobility and finger grip strength of the children were significantly improved after treatment (P<0.05). telephone follow-up after surgery showed that 1 case of secondary angular deformity and 1 case of residual phalangeal osteophyte appeared, and both above 2 children recovered well after secondary surgery.

**Conclusion:** Wassel classification-assisted polydactyly replantation for children with congenital thumb deformity has a significant clinical effect, which is conducive to promoting the recovery of children’s thumb function and can effectively improve children’s finger mobility and finger level.

## 024 | A randomized controlled study on the treatment of perimenopausal insomnia with the acupuncture method of “regulating liver and nourishing blood”

Zixuan Zhao^1^, Guangzhong Du^2^, Qingchen Zhou^2^

^*1*^*Acupuncture Moxibustion and Tuina department, The Affiliated Hospital of Shandong Institute of Traditional Chinese Medicine, Jinan, China,* ^*2*^*Acupuncture Moxibustion and Tuina department, Qilu Hospital of Shandong University, Jinan, China*

**Objective:** This study aims to verify the clinical efficacy of the “regulating liver and nourishing blood” acupuncture method in treating insomnia during perimenopause.

**Methods:** 84 patients were randomly divided into an experimental group (EG)and a control group (CG). The CG was given alprazolam tablets 0.4mg, once a night, for 4 weeks, The EG used the “regulating liver and nourishing blood” acupuncture. The treatment time is 30 minutes each time, once a day, for a total of 4 weeks. Then compare the changes in PSQI, KI, MENQOL, SDS, SAS scores and serum E2, LH, FSH, and AMH levels between two groups of patients before and after treatment.

**Results:** After 4 weeks of treatment, the scores of PSQI, KI, MENQOL, SDS, and SAS in the EG were significantly lower than those before treatment and those in the CG (P<0.05). Serum E2 and AMH levels were significantly higher than those before treatment and those in the CG. FSH levels were significantly lower than those in CG (P<0.05), while LH levels showed no significant change (P>0.05).

**Conclusion:** Acupuncture with “regulating liver and nourishing blood” method can improve the sleep quality, alleviate anxiety and depression, enhance the quality of life, and regulate hormonal imbalances in perimenopausal women.

**Acknowledgements:** Shandong Province Traditional Chinese Medicine Science and Technology Project 2021Q119. National Key R&D Program 26010105482301.

## 025 | Effects of remifentanil dose on hemodynamics stability and cognitive function in patients with acute brain injury during total intravenous anesthesia

Liankai Yang

*Panzhihua Central Hospital, Panzhihua, China*

**Objective:** To observe the effects of remifentanil dose on hemodynamics stability and cognitive function in patients with acute brain injury (ABI) during total intravenous anesthesia (TIVA).

**Methods:** A total of 65 patients with ABI undergoing TIVA in the hospital were retrospectively enrolled between January 2022 and January 2025. All were given 6mg/(kg·min) propofol and remifentanil for continuous anesthesia. According to different remifentanil doses, they were divided into group A [n=22, 0.1μg/(kg·min)], group B [n=23, 0.25μg/(kg·min)] and group C [n=20, 0.5μg/(kg·min)]. The changes of cerebral hemodynamics indexes [carotid blood flow (Qmean), carotid flow velocity (Vmean), pulsatility index (PI), resistance index (RI)], heart rate (HR) and mean arterial pressure (MAP) before anesthesia induction (T_0_), after tracheal intubation (T_1_), immediately after skin incision (T_2_) and at 30min after the start of surgery (T_3_) in the three groups were compared. The cognitive function was evaluated by mini-mental state examination (MMSE) before and after surgery. The postoperative recovery and adverse reactions were statistically analyzed.

**Results:** Qmean and Vmean at T_2_ and T_3_ were lower than those at T_0_ and T_1_ in the three groups, and which were higher in groups B and C than group A (*P*<0.05). PI and RI at T_2_ and T_3_ were higher than those at T_0_ and T_1_ in the three groups, and which were lower in groups B and C than group A (*P*<0.05). At T_2_ and T_3_, there was no significant difference in Qmean, Vmean, PI or RI between group B and group C (*P*>0.05). At T_2_ and T_3_, levels of HR and MAP in groups B and C were lower than those in group A (*P*<0.05). At 1d after surgery, MMSE score in group C was lower than that in groups A and B *P*<0.05). The incidence of cognitive function decline in group A and group B was 4.55% and 8.70%, lower than that in group C (35.00%, *P*<0.05). In addition, recovery time and total incidence of adverse reactions in groups A and B were lower than those in group C (*P*<0.05).

**Conclusion:** 0.25μg/(kg·min) remifentanil has better anesthesia quality in ABI patients during TIVA, which can improve hemodynamics stability, promote the recovery of cognitive function and is beneficial to improve postoperative recovery and the occurrence of adverse reactions.

## 026 | Clinical application of pit and fissure sealant in oral cavity and study on its preventive effect on dental caries

Yan Zheng

*School of Dental Medicine, Dalian University, Dalian, China*

**Objective:** The aim of this study is to explore the effective application of pit and fissure sealing surgery in oral clinical practice, and evaluate its practical effect on preventing dental caries in 240 patients with dental caries, providing scientific basis for optimizing oral caries prevention strategies.

**Methods:** Select the research subjects. 240 patients diagnosed with high-risk factors for dental caries (such as deep pit and fissure, caries tendency, etc.) through oral examination, aged 6-15 years, were randomly divided into an experimental group and a control group, with 120 patients in each group Implementation of intervention methods. Patients in the experimental group were treated with pit and fissure sealing surgery to prevent dental caries. Firstly, clean and etch the patient’s dental cavities and grooves, then apply sealant and cure them with a light curing lamp. The control group patients only received routine oral health education, including correct brushing methods, dietary precautions, etc., and did not undergo pit and fissure sealant surgery Determine observation indicators. Follow up examinations were conducted on two groups of patients at 6, 12, and 24 months after intervention. The observation indicators mainly include the number of newly developed dental caries and the degree of caries development (evaluated using the International Caries Detection and Assessment System).

**Results:** (1) Number of newly developed dental caries. After 6 months of intervention--the experimental group had 3 new dental caries (2.5%), while the control group had 15 new dental caries (12.5%), and the difference between the two groups was statistically significant (P<0.05). After 12 months of intervention, the number of newly developed dental caries in the experimental group was 7 (5.83%), while in the control group it was 32 (26.67%), with a significant difference (P<0.05). At 24 months of intervention, the number of newly developed dental caries in the experimental group was 12 (10%), while in the control group it was 62 (51.67%). (2) Development degree of dental caries--According to the International Caries Detection and Assessment System, the experimental group showed relatively delayed development of caries. After 24 months of intervention, only 15.8% of patients in the experimental group had grade III caries (significant caries), compared to 43.3% in the control group.

**Conclusion:** Groove closure surgery has important application value in oral clinical practice. The results of this study showed that pit and fissure closure surgery can significantly reduce the number of new dental caries in high-risk populations (such as the 240 patients in this study). Compared with pure oral health education, pit and fissure sealants effectively prevent bacteria and food residues from entering the dental pit and fissure, thereby reducing the risk of dental caries long term continuous prevention has a significant effect. During the 24 months observation period, the advantages of pit and fissure closure became increasingly apparent over time. The difference in the number of newly developed dental caries between the experimental group and the control group continues to increase, and there are also significant differences in the degree of caries development. This indicates that pit and fissure sealant is not only a short-term preventive measure for dental caries, but also plays an irreplaceable role in reducing the development of dental caries in the long run Implications for dental caries prevention strategies. The results of this study suggest that pit and fissure sealants should be actively promoted in the prevention of dental caries. Especially for high-risk groups of dental caries, pit and fissure sealant can be used as an effective early prevention method. Combined with other preventive measures such as oral health education, it can build a more comprehensive dental caries prevention system and improve oral health.

## 027 | Analysis of clinical characteristics of short-term postoperative neurological abnormalities after cardiac surgery

Ning Gai, Kaiqiang Yang, Feng Wang, Shengjun Dong, Engang Wu

*Department of Cardiovascular Surgery, Binzhou Medical University Hospital, Binzhou, China*

**Objective:** The project is to analyze the clinical characteristics of short-term postoperative neurological abnormalities after cardiac surgery in depth and improve this complication’s cognition and coping ability.

**Methods:** Data of 500 patients who underwent cardiac surgery in our hospital from January 2018 to December 2023 and survived for more than 24 hours after surgery were retrospectively collected. The patients’ preoperative basic diseases, such as hypertension, diabetes, cerebrovascular disease history, the type of surgery, such as coronary artery bypass grafting, heart valve replacement, etc., and information such as extracorporeal circulation time, were recorded. After surgery, the patient’s neurological manifestations were closely observed through neurological physical examination, head CT or MRI imaging examination, and neuroelectrophysiological examination, and the occurrence and type of abnormalities were clarified, and the data were analyzed using statistical software.

**Results:** Among the 500 patients, 60 had neurological abnormalities in the short term after surgery, with an incidence of 12%. The symptoms were diverse, with delirium being the most common, with 35 cases (58.3%), manifested as confusion, agitation, etc., followed by 15 cases (25%) of stroke, including ischemic and hemorrhagic, and 8 cases of peripheral neuropathy (13.3%) and 2 cases of epilepsy (3.4%). Univariate analysis showed that the incidence of postoperative neurological abnormalities was significantly increased in patients aged ≥65 years, with a history of cerebrovascular disease before surgery, with extracorporeal circulation for more than 120 minutes, and with heart valve replacement (all P<0.05). Multivariate Logistic regression analysis showed that prolonged extracorporeal circulation time (OR = 3.56, 95% CI: 1.89 - 6.69) and a history of cerebrovascular disease before surgery (OR = 2.85, 95% CI: 1.42 - 5.73) were independent risk factors for postoperative neurological abnormalities.

**Conclusion:** Short-term neurological abnormalities after heart disease surgery are not uncommon, and the symptoms are complex. Extracorporeal circulation time and preoperative history of cerebrovascular disease are key risk factors. Clinicians should pay attention to this and strengthen preoperative evaluation and postoperative monitoring of high-risk patients, detect and intervene as early as possible, and improve patient prognosis.

## 028 | Clinical perinatal management of fetuses with high-risk congenital pulmonary airway malformations

Jian Kang^1^, Tingting Xu^2^, XiaoLi Tian^3^, Lu Li^4^

^*1*^*Department of Pediatrics, Shenyang Children’s Hospital, Shenyang, China,* ^*2*^*Department of medicine.Tianjin Corps Hospitai of Chinese Armed Police Force, Tianjin, China,* ^*3*^*Medical Department,Shenyang Children’s Hospital, Shenyang, China,* ^*4*^*Neonatal severe disease The Second Affiliated Hospital of Shaanxi University of Chinese Medicine, Xixian New District Central Hospital, Shaanxi, China*

**Objective:** The paper explores effective perinatal management strategies for high-risk congenital pulmonary airway malformation (CPAM) fetuses and improves the prognosis of fetuses and neonates.

**Methods:** We reviewed 50 cases of high-risk CPAM fetuses diagnosed in our hospital from January 2018 to December 2023. With the help of fetal ultrasound and MRI examinations, high-risk criteria were defined based on lesion size, blood flow status, and the presence or absence of fetal edema. Multidisciplinary collaborative management was adopted during the perinatal period. Prenatal ultrasound and MRI were used for close monitoring, and glucocorticoids were used to promote fetal lung maturity when appropriate, for high-risk fetuses such as fetal edema, intrauterine interventions such as fetoscopic laser coagulation were considered. The timing and method of delivery were determined based on the fetal condition and gestational age. The newborns were immediately transferred to the intensive care unit after delivery for respiratory support and anti-infection treatment, and the relationship between management measures and outcomes was analyzed.

**Results:** After multidisciplinary management, 45 of the 50 cases were successfully delivered, and the neonatal survival rate was 80% (36/45). Prenatal monitoring helped to adjust interventions promptly. Among the 10 fetuses who received intrauterine interventions, 6 of them improved. Timely use of glucocorticoids reduced the incidence of neonatal respiratory distress syndrome. Reasonable postpartum respiratory support and anti-infection are of great significance to neonatal survival.

**Conclusion:** The multidisciplinary collaborative perinatal management model can significantly improve the prognosis of high-risk CPAM fetuses and neonates and increase survival through accurate prenatal monitoring, timely intrauterine intervention, reasonable delivery planning, and active postpartum treatment.

## 029 | Clinical comparative study of laparoscopic and traditional open peritoneal dialysis catheterization

Han Song^1^, Juan Tu^2^, Huarong Li^2^, Hongyang Wang^3^

^*1*^*Department of Nursing Department, Capital Institute of Pediatrics, Beijing, China;* ^*2*^*Department of Nephrology, Capital Institute of Pediatrics, Beijing, China;* ^*3*^*Department of Urology, Capital Institute of Pediatrics, Beijing, China*

**Objective:** The paper compares the effects of laparoscopic and traditional open peritoneal dialysis catheterization to help optimize catheterization methods clinically.

**Methods:** 120 patients with end-stage renal disease who required peritoneal dialysis catheterization in our hospital from January 2022 to December 2024 were selected and divided into laparoscopic and open groups according to the random number table method. The laparoscopic group underwent laparoscopic catheterization, and the open group used traditional open catheterization. The operation times, intraoperative blood loss, first postoperative dialysis, and hospitalization times were recorded for the two groups. The follow-up was 6 months, and the incidence of catheter-related complications (infection, displacement, blockage, etc.) was counted. The Kidney Disease Quality of Life Short Form (KDQOL-SF) was used to evaluate patients’ quality of life. SPSS 22.0 software was used for analysis. The measurement data were expressed as mean ± standard deviation, and the independent sample t-test was performed. The enumeration data were expressed as a rate (%), and the chi-square test was used. The difference was considered statistically significant when P<0.05.

**Results:** The operation time of the laparoscopic group [(65.2±10.5) min] was longer than that of the laparotomy group [(50.8±8.3) min, P<0.05], but the intraoperative blood loss [(25.6±5.2) mL] was less than that of the laparotomy group [(55.3±12.6) mL, P<0.05]. The first postoperative dialysis [(2.3±0.6) d] and hospital stay [(5.5±1.2) d] of the laparoscopic group were shorter than those of the laparotomy group [(3.5±0.8) d, (8.2±1.5) d, P<0.05]. During the follow-up period, the complication rate in the laparoscopic group (10.0%) was lower than that in the laparotomy group (25.0%, P<0.05), and the quality-of-life score was higher (P<0.05).

**Conclusion:** Compared with traditional laparotomy catheterization, laparoscopic catheterization has a slightly longer operation time, but it has less intraoperative bleeding, faster recovery, and fewer complications. These can improve patients’ quality of life and make it a more advantageous peritoneal dialysis catheterization method.

**Acknowledgements:** This work was supported by the Beijing Municipal Administration of Hospitals Incubating Program (NO. PX20231306).

## 030 | Clinical study on the effect of pit and fissure sealant combined with fluoride protective paint in preventing dental caries in children

Yan Zheng

*School of Dental Medicine, Dalian University, Dalian, China*

**Objective:** Compare the clinical effects of using pit and fissure sealant alone, fluoride protective paint alone, and their combined application to prevent dental caries in children, providing a basis for the selection of clinical dental caries prevention measures.

**Methods:** Select 800 children aged 3-8 who meet the inclusion criteria and randomly divide them into three groups. Groove closure surgery group: 268 cases of groove closure surgery were performed on the first permanent molar of children, with 1072 teeth closed. Fluoroprotective paint group (Fluoroprotective paint group): Fluoroprotective paint was applied to the first permanent molars in 270 cases, with 1083 teeth coated. Combination group of pit and fissure sealant and fluoride protective paint: 262 cases were treated with pit and fissure sealant first, followed by fluoride protective paint coating. A total of 1048 teeth were sealed and coated. Follow up at 6, 12, and 18 months after treatment to check for the occurrence of dental caries. The pit and fissure sealant surgery is performed according to standard operating procedures, with strict adherence to procedures such as moisture isolation, acid etching, and resin coating. Fluorine protective paint adopts standard coating methods to ensure uniform coating.

**Results:** At the follow-up visit in the sixth month, it was found that the incidence of dental caries in the fissure sealant group was 88.6% (950/1072), The incidence of dental caries in the fluoride protective paint group was 87.2% (945/1083), The incidence of dental caries was 94.2% (987/1048) in the group of pit and fissure sealant combined with fluoride protective paint. There was no significant difference in the incidence of caries between the fissure sealant group and the fluoride protective paint group (P>0.05). The fissure sealant combined with fluoride protective paint group had a significantly higher incidence of caries compared to the other two groups (P<0.05).

At the 12-month follow-up, the incidence of dental caries in the pit and fissure sealant group was 76.1% (815/1072), The fluorine protective paint group accounts for 73.8% (798/1083), The combination of pit and fissure sealant and fluoride protective paint group accounted for 85.6% (899/1048). There was no significant difference in the incidence of caries between the fissure sealant group and the fluoride protective paint group (P>0.05). The fissure sealant combined with fluoride protective paint group had a significantly higher incidence of caries compared to the other two groups (P<0.05).

At the 18th month follow-up, the incidence of dental caries in the pit and fissure sealant group was 63.5% (679/1072), The fluorine protective paint group accounts for 61.2% (662/1083), The combination of pit and fissure sealant and fluoride protective paint group accounted for 74.9% (785/1048). There was no significant difference in the incidence of caries between the fissure sealant group and the fluoride protective paint group (P>0.05). The fissure sealant combined with fluoride protective paint group had a significantly higher incidence of caries compared to the other two groups (P<0.05).

**Conclusion:** The combination of pit and fissure sealant and fluoride protective paint has shown good clinical efficacy in preventing dental caries in children, and its effectiveness in preventing dental caries is significantly better than that of single pit and fissure sealant or fluoride protective paint. Groove closure surgery can effectively seal the molars’ grooves and prevent the retention of bacteria and food residues in the grooves, Fluoride protective paint can enhance the acid resistance of dental enamel and inhibit bacterial growth. The combined application of the two can synergistically exert anti caries effects from different mechanisms, and can continuously provide good anti caries effects in long-term follow-up. This joint method has important promotional value in clinical prevention of dental caries in children, which helps to take more effective measures to reduce the incidence of dental caries in children and protect their oral health.

## 031 | Research on Medical Image Processing Based on Deep Learning Technology

Ying Shi^1^, Guoyue Liu^2^, Xiaohong Hao^1^

^*1*^*Department of Basic Courses, Suzhou City University, Suzhou, China;* ^*2*^*Research and Development Centre, Insnex Technologies Co., Ltd, Suzhou, China*

**Objective:** The main purpose of this study is to explore the application of deep learning technology in medical image processing. Through training and learning a large amount of medical image data, efficient and accurate image analysis and diagnostic models are constructed to provide more accurate and rapid diagnostic support for the medical field.

**Methods:** In terms of data collection and preprocessing, this study collected a total of approximately 10000 medical image data, including X-rays, CT, MRI, etc., from different medical institutions covering various types of diseases. After organizing the data, a series of preprocessing operations such as standardization, normalization, and data enhancement were carried out on these images to improve the training effectiveness and generalization ability of the model. In terms of model construction and training, this study selected Convolutional Neural Networks (CNN) from deep learning as the basic model and made a series of improvements and optimizations based on the characteristics of medical images. The model structure includes multiple convolutional layers, pooling layers, and fully connected layers, and is trained using cross entropy loss function and stochastic gradient descent algorithm. The training process is conducted on deep learning frameworks such as TensorFlow or PyTorch, using graphics processing units to accelerate computation. In terms of evaluation and validation, the dataset was divided into training and testing sets in an 8:2 ratio in this study. Evaluate the performance of the model using multiple indicators such as accuracy, recall, specificity, and F1 score. Meanwhile, the stability and reliability of the model are further verified through cross validation methods.

**Results:** Firstly, the accuracy of disease diagnosis has significantly improved. On the test set, the model achieved an accuracy rate of 92.5% for the diagnosis of common diseases including lung diseases, cardiovascular diseases, etc. Among them, for the diagnosis of lung diseases, the accuracy rate is as high as 95.3%, the recall rate is 93.8%, the specificity is 94.8%, and the F1 score is 0.94, For the diagnosis of cardiovascular disease, the accuracy rate is 90.2%, the recall rate is 89.5%, the specificity is 90.9%, and the F1 score is 0.90. Secondly, the accuracy of locating the lesion area is high. The model can not only accurately diagnose diseases, but also effectively locate lesion areas. On a large-scale test set, the average accuracy of lesion localization reached 88.7%, with an average intersection to union ratio of 0.76. For example, in brain medical images, the localization accuracy of brain tumors is as high as 91.2%, providing clinical doctors with more accurate lesion information.

**Conclusion:** In this study, a medical image processing model constructed using deep learning technology achieved good results in disease diagnosis and lesion area localization. Through training and optimization on large-scale datasets, the model can accurately identify multiple diseases and locate lesion areas, providing powerful diagnostic support for the medical field. Overall, the application of deep learning technology in medical image processing has enormous potential. In the future, the application scope of the model can be further expanded, such as processing more types of diseases and medical image modalities. In short, the combination of deep learning technology and medical image processing will bring new opportunities and challenges to the development of the medical industry.

## 032 | Research on Medical Data Mining Based on Association Rule Algorithm

Ziyi Yang, Yuxiao Liu

*School of Computer Engineering, Hubei University of Arts and Science, Xiangyang, China*

**Objective:** In the medical field, there is a large amount of electronic medical records, test reports, test results and other data, which contain rich information, but the information is often not fully explored and utilized. This study aims to use association rule algorithms to identify potential relationships between variables in massive medical data, such as the association between a certain disease and symptoms, examination results, and treatment plans, providing a basis for clinical diagnosis, disease prevention, and treatment plan selection. In this study, association rule algorithms were used to mine medical data from 1200 medical records, hoping to discover potential associations between diseases and symptoms, examinations, treatments, and other aspects, thereby helping to improve the effectiveness of clinical diagnosis and treatment decisions.

**Methods:** Firstly, data collection and preprocessing. This study collected 1200 electronic medical records, including basic information, symptoms, diagnostic results, examination results, and treatment plans of patients. Clean the data, remove missing and erroneous values, standardize various medical terms, such as unifying different symptom expressions into standardized medical names. Then, build a transaction database. In this process, each medical record is treated as a transaction, and the symptoms, examinations, and treatments in the medical record are treated as items to build a transaction database. Finally, there is association rule mining. The main approach is to use the Apriori algorithm for association rule mining, with a minimum support of 0.05 and a minimum confidence of 0.6, to extract frequent itemsets and association rules.

**Results:** Firstly, frequent itemset discovery. During this process, a total of 12 frequent 2-itemsets were excavated, for example, it was found that the frequency of “hyperlipidemia” and “hypertension” occurring simultaneously was relatively high, with a support score of 0.12. Next is the generation of association rules. Eight valid association rules have been generated. Some representative rules are as follows: ① The confidence level of “palpitations → coronary heart disease” is 0.70, indicating that 70% of patients with palpitations are diagnosed with coronary heart disease. ② The support score for ‘cough and fever → pneumonia’ is 0.08, with a confidence level of 0.75, indicating that 75% of patients are diagnosed with pneumonia when cough and fever occur simultaneously. Finally, there is the exploration of treatment plans. The results showed an association rule between “aspirin+clopidogrel” and “post stent implantation” with a confidence level of 0.80, indicating that a large proportion of patients use these two drugs for treatment after stent implantation.

**Conclusion:** Association rule algorithms can effectively mine potential associations between diseases, symptoms, examinations, and treatments from a large amount of medical record data. This study has discovered many clinically significant associations, such as specific symptom combinations providing clues for disease diagnosis, and the close relationship between certain treatment options and specific diseases. These mining results help clinical doctors make faster and more accurate diagnoses. For example, when a patient experiences “palpitations” symptoms, doctors will highly suspect coronary heart disease and conduct further examinations. In terms of treatment decision-making, the correlation results can provide reference for doctors to recommend more scientific and reasonable treatment plans. For patients after stent implantation, the combination therapy of aspirin and clopidogrel can be given priority consideration.

## 033 | Research on Topological Data Analysis for Feature Extraction and Classification in Medical Imaging

Meili Zhang^1^, Hongmei Pei^1^, Weili Liu^1^, Daizhen Wu^2^, Jiaming Cheng^2^

^*1*^*Department of the foundation, Dalian Naval Academy, Dalian, China;* ^*2*^*Cadet team, Dalian Naval Academy, Dalian, China*

**Objective:** The objective of this study is to specifically explore the application effects of Topological Data Analysis (TDA) in the extraction and classification of medical image features, and to assess its capabilities in enhancing the accuracy and efficiency of medical image recognition. The research will focus on analyzing the performance of TDA in identifying tumors, blood vessels, and other key biomarkers in medical images.

**Methods:** In this study, we employed methods of Topological Data Analysis (TDA), with Persistent Homology as the core tool. By constructing Vietoris-Rips complexes (VR complexes), we transformed medical image data into topological spaces to extract topological features of the images. This approach allowed us to discover hidden topological structures in high-dimensional data and utilize them for feature extraction and classification of images. We used a series of publicly available medical image datasets, including MRI and CT scan images, to train and test our model.

**Results:** The experimental results indicate that TDA shows significant potential in the extraction and classification of medical image features. In the task of tumor segmentation in brain MRI images, our method achieved an accuracy rate of 85%, which is a 10% improvement over traditional machine learning methods. In the task of nodule detection in lung CT images, the sensitivity of the TDA method reached 90%, and the specificity reached 95%, demonstrating the potential of TDA in enhancing the robustness of models. Additionally, we found that TDA can identify subtle structural changes that are difficult for traditional methods to detect, which is of great significance for the diagnosis of early-stage lesions.

**Conclusion:** This study concludes that Topological Data Analysis (TDA) has significant application value in the field of feature extraction and classification in medical imaging. Our research results show that TDA can significantly improve the robustness of models and maintain the accuracy of recognition when dealing with complex medical image data. Therefore, TDA provides a new and powerful analytical tool for the field of medical image recognition, which is expected to play a greater role in future medical image processing and pattern recognition tasks. Our research not only lays the foundation for further exploration of TDA in medical image analysis but also provides new ideas for improving the accuracy and efficiency of medical image recognition technology.

**Acknowledgements:** This research was financially supported by the scientific research and development fund project of Dalian Naval Academy.

## 034 | Study on the contraceptive effect of placing Manyue Le at different times after induced abortion surgery

Zhuo Chen

*Jinhua Municipal Centeral Hospital Medical Group, Jinhua, China*

**Objective:** The main purpose is to analyze and evaluate the contraceptive effectiveness, safety, and impact on female reproductive health of 92 cases of artificial abortion treated with different time periods of placement of Manyue Le, hoping to provide scientific basis for the rational selection and application of clinical contraceptive measures.

**Methods:** 92 women who underwent induced abortion surgery in our hospital and had contraceptive needs were randomly divided into three groups according to different treatment methods: observation group A, observation group B, and conventional group. Observation group A (n=31): immediate placement of Manyue Le after induced abortion surgery, Observation of Group B (n=30): Manyue Le was placed 3-7 days after the resumption of menstruation, one month after induced abortion, Conventional group (n=31): Manyue Le was placed 2 months after induced abortion and 3-7 days after menstruation resumed. Three groups of patients were regularly followed up for 12 months after placement of Manyue Le. The contraceptive failure (such as unintended pregnancy), adverse reactions (such as irregular vaginal bleeding, changes in menstrual flow, etc.), and patient satisfaction were recorded for each group of patients.

**Results:** 1. Contraceptive effect: During the follow-up period of Group A, there was no unexpected pregnancy, and the success rate of contraception reached 100%. During the follow-up period, there was one unexpected pregnancy in Group B, and the success rate of contraception reached 96.7% (29/30). During the follow-up period, there were 2 cases of unintended pregnancy in the routine group, and the success rate of contraception reached 93.5% (29/31). The success rate of contraception in Group A was significantly higher than that in Group B and the conventional group, with statistical significance (P<0.05).

2. Adverse reaction occurrence: All three groups of patients experienced varying degrees of irregular vaginal bleeding after placement of Manyue Le. The incidence rate of observation group A reached 48.4% (15/31), observation group B reached 50.0% (15/30), and the conventional group reached 51.6% (16/31). There was no statistically significant difference between the groups (P>0.05). The incidence of reduced menstrual flow in observation group B and conventional group was 83.3% (25/30) and 83.9% (26/31), respectively, both higher than observation group A’s 64.5% (20/31), and the difference was statistically significant (P<0.05).

3. Patient satisfaction: The satisfaction rate of patients in observation group A reached 90.3% (28/31), observation group B reached 80.0% (24/30), and the conventional group reached 77.4% (24/31). The satisfaction rate of patients in observation group A was significantly higher than that in observation group B and the conventional group, with statistical significance (P<0.05).

**Conclusion:** The best contraceptive effect is to immediately place Manyue Le after induced abortion, which avoids the risk of unintended pregnancy in a short period of time and has high patient satisfaction. Although there was not much overall difference in the incidence of adverse reactions among the three groups, immediate placement after surgery may be more advantageous in exerting the regulatory effect of Manyue Le on menstruation, as evidenced by the reduction in menstrual flow. In practical clinical practice, placing Manyue Le immediately after induced abortion is a recommended contraceptive method for eligible patients. It can improve the effectiveness of contraception, reduce the health risks faced by women due to unintended pregnancies, and increase patient acceptance and satisfaction, providing better protection for women’s reproductive health.

## 035 | Transitional nursing plan and effectiveness evaluation for elderly patients with permanent colostomy

Youmin Liu, Lijuan Zhang

*The first affiliated hospital of soochouw university, General surgery, Suzhou, China*

**Objective:** In today’s rapidly aging population, the number of elderly patients with permanent colostomy is gradually increasing. These patients face many physiological and psychological challenges during the transition period, and will face many difficulties in adapting to new physical conditions and lifestyles. To address this issue, this article observes and evaluates the effectiveness of targeted nursing interventions for elderly patients with permanent colostomy during the transition period, hoping to provide scientific basis for clinical nursing and improve the quality of life of patients during the transition period.

**Methods:** 90 elderly patients with permanent colostomy were selected as the research subjects and randomly divided into a nursing intervention group and a routine nursing group, with 45 patients in both groups. The routine care group provides routine care, including basic vital sign monitoring, wound care, etc. The nursing intervention group implemented comprehensive transitional nursing interventions on the basis of routine nursing. The specific measures are as follows: Before surgery, through one-on-one communication, holding health lectures, and other methods, explain in detail the relevant knowledge of colostomy surgery to patients and their families to alleviate their anxiety and fear, Assist patients in preparing their intestines and arranging their diet reasonably. In the early postoperative period, closely observe the condition of the stoma and surrounding skin, and promptly handle any abnormal situations, Guide patients and their families to perform correct ostomy care procedures, such as cleaning, replacing ostomy bags, etc, Develop personalized dietary plans based on the patient’s recovery status and guide them on a reasonable diet. After discharge, establish a long-term follow-up mechanism to regularly understand the patient’s condition through phone calls, text messages, home visits, and other means, Provide continuous guidance on stoma care for patients, including the selection and replacement of stoma supplies, Organize patient exchange meetings, encourage patients to share their experiences and insights with each other, and enhance their confidence in recovery.

**Results:** After intervention, the anxiety and depression scores of patients in the nursing intervention group were significantly lower than those in the conventional nursing group (P<0.05), indicating a significant improvement in the psychological state of patients in the nursing intervention group. In terms of self-care ability, patients in the nursing intervention group had significantly better mastery of stoma nursing skills than those in the conventional nursing group (P<0.05), and were able to complete stoma nursing operations more proficiently and accurately. In addition, the incidence of stoma related complications in the nursing intervention group was significantly lower than that in the conventional nursing group (P<0.05), and the quality of life score was significantly higher than that in the conventional nursing group (P<0.05), indicating that patients in the nursing intervention group can better adapt to life after intestinal stoma in both physical and psychological states.

**Conclusion:** This study shows that comprehensive, systematic, and targeted nursing interventions for elderly patients with permanent colostomy during the transition period have a good effect, effectively reducing their psychological burden, improving their self-care ability, reducing the incidence of stoma related complications, and enhancing their quality of life. This nursing intervention model is worth widely applying and promoting in clinical practice, providing better nursing services for elderly patients with permanent colostomy, helping them better adapt to new living conditions and return to normal life.

## 036 | Intervention effect of mental health education based on mindfulness therapy on patients with anxiety disorders

Yixin Zhang, Xiaobin Ding, Xiangling Tu

*School of Psychology, Northwest Normal University, Lanzhou, China*

**Objective:** This study aims to explore the intervention effect of mental health education based on mindfulness therapy on patients with anxiety disorders and provide a scientific basis for optimizing the clinical intervention program for anxiety disorders.

**Methods:** This paper adopts a randomized controlled experimental design. 120 patients who met the diagnostic criteria for anxiety disorders in the Diagnostic and Statistical Manual of Mental Disorders, Fifth Edition (DSM-5) were selected and randomly divided into an intervention group and a control group, with 60 cases in each group. The control group received conventional drug treatment and health education. The intervention group conducted an 8-week, 90-minute mindfulness therapy mental health education course twice weekly, covering mindfulness breathing, body scanning, mindfulness meditation, and other training. The Hamilton Anxiety Rating Scale (HAMA), Self-Rating Depression Scale (SDS), and Comprehensive Quality of Life Assessment Questionnaire (GQOLI-74) were used to evaluate the anxiety and depression levels and quality of life of the two groups of patients before, after, and 3 months after the intervention.

**Results:** After the intervention and 3 months after the intervention, the HAMA and SDS scores of the intervention group were significantly lower than those of the control group (P<0.05), and the scores of each dimension of quality of life in the intervention group were significantly higher than those in the control group (P<0.05). In addition, the improvement of anxiety symptoms in the intervention group remained stable during the follow-up period.

**Conclusion:** Mindfulness-based mental health education can effectively relieve the anxiety and depression symptoms of patients with anxiety disorders, significantly improve their quality of life, and the intervention effect has a certain degree of persistence, which can be used as an effective supplementary means for the comprehensive treatment of anxiety disorders.

## 037 | Analysis of influencing factors of complications after laryngeal cancer surgery

Yinping Zeng, Xiaofeng Wang, Tingting Duan, Jiajun Huang

*Department of Otorhinolaryngology Head and Neck Surgery, The First Affiliated Hospital of Hainan Medical University, Haikou, China*

**Objective:** This study aims to accurately identify the influencing factors of postoperative complications of laryngeal cancer and provide a solid basis for the clinical formulation of efficient prevention and intervention strategies.

**Methods:** A retrospective analysis was conducted on the clinical data of 300 patients with laryngeal cancer who underwent surgical treatment in our hospital from January 2018 to December 2023. SPSS 25.0 statistical software was used to first perform univariate analysis to screen out factors that may be related to postoperative complications. The factors with statistical significance in the univariate analysis were then included in the multivariate logistic regression model for further exploration.

**Results:** Univariate analysis showed that factors such as patient age (complication rate was 45.0% in the group ≥60 years old and 28.6% in the group <60 years old, P <0.05), tumor stage (complication rate was 22.2% in stage I-II and 56.7% in stage III-IV, P <0.05), whether or not there was chronic obstructive pulmonary disease (complication rate was 55.0% in the combined group and 30.8% in the uncombined group, P <0.05), and operation time (complication rate was 48.1% in the group >3 hours and 26.4% in the group ≤3 hours, P <0.05) were significantly associated with the incidence of complications after laryngeal cancer surgery. Multivariate analysis showed that advanced tumor stage (Ⅲ-Ⅳ stage) (OR = 3.25, 95% CI: 1.86 - 5.72), combined chronic obstructive pulmonary disease (OR = 2.88, 95% CI: 1.53 - 5.42) and longer operation time (>3 hours) (OR = 2.56, 95% CI: 1.37 - 4.79) were independent risk factors for postoperative complications of laryngeal cancer.

**Conclusion:** Tumor stage, combined chronic obstructive pulmonary disease, and operation time are important influencing factors for postoperative complications of laryngeal cancer. In clinical practice, for high-risk patients with advanced tumors, combined chronic obstructive pulmonary disease, and long operation times, perioperative management should be strengthened, and surgical plans should be further optimized to reduce the risk of postoperative complications and improve the treatment effect and quality of life of patients.

**Acknowledgements:** This work was supported by the Effect of SMG-1 on radiosensitivity of head and neck squamous cell carcinoma and its signaling pathway (NO.820MS141), Regulation of immune microenvironment of head and neck squamous cell carcinoma by SERS probes via RIG-NFkB axis reprogramming TAM (NO.824QN381).

## 038 | Correlation analysis between postoperative delirium and perioperative sleep disorders in elderly patients undergoing lower limb orthopedic surgery

Zihan Ma

*Department of Anesthesiology, The Affiliated People’s Hospital of Ningbo University, Ningbo, China*

**Objective:** This paper analyzes the correlation between postoperative delirium and perioperative sleep disorders in elderly patients undergoing lower limb orthopedic surgery and provides a basis for clinical prevention and treatment.

**Methods:** 150 elderly patients who underwent lower limb orthopedic surgery in our hospital from January 2022 to December 2023 were selected. The Pittsburgh Sleep Quality Index (PSQI) was used to evaluate the patient’s sleep status before surgery, and the Confuscious Assessment Method (CAM) was used to diagnose the occurrence of delirium after surgery. The sleep parameters of patients during the perioperative period (1 day before surgery, 1 day after surgery, and 3 days after surgery) were recorded by polysomnography, including total sleep time, sleep efficiency, and number of awakenings. Statistical software was used to analyze the correlation between postoperative delirium and various indicators of perioperative sleep disorders.

**Results:** Among the 150 patients, 25 cases of postoperative delirium occurred, with an incidence of 16.7%. The preoperative PSQI score of patients with delirium was significantly higher than that of patients without delirium (P<0.05). Perioperative sleep monitoring showed that patients’ total sleep time and sleep efficiency in the delirium group were lower than those in the non-delirium group. The number of awakenings was more than that in the non-delirium group, and the differences were statistically significant (P<0.05). Multivariate Logistic regression analysis showed that poor preoperative sleep quality (OR=2.56, 95% CI: 1.23-5.32), short total postoperative sleep time (OR=3.15, 95% CI: 1.67-5.94) and more awakenings (OR=2.89, 95% CI: 1.48-5.65) were independent risk factors for postoperative delirium in elderly patients undergoing lower limb orthopedic surgery.

**Conclusion:** Postoperative delirium in elderly patients undergoing lower limb orthopedic surgery is closely related to perioperative sleep disorder. Poor preoperative sleep quality and postoperative sleep disorders will increase the risk of postoperative delirium. Clinical attention should be paid to perioperative sleep management to reduce the incidence of postoperative delirium.

## 039 | Analysis of influencing factors of short-term prognosis of Alzheimer’s disease and care countermeasures

Xiaohong Liu, Xiaoying Lai, Bei Yang, Ruyin Lian, Meixiang Zeng

*Department of Geriatrics - the Third Hospital Of Longyan, Longyan, China*

**Objective:** This paper analyzes the factors affecting the short-term prognosis of Alzheimer’s disease and formulates care strategies to improve the patient’s condition.

**Methods:** From January 2022 to December 2023, 150 patients with AD were selected. Data such as age, gender, education level, course of disease, underlying diseases, cognitive function (MMSE score), and daily living activities (BI score) were collected. Follow-up was performed for 6 months to evaluate the prognosis. After univariate screening, multivariate logistic regression was used to determine the independent influencing factors, and care strategies were formulated accordingly.

**Results:** The univariate analysis showed that patients aged ≥75 years, low education level, long course of disease, combined with hypertension or diabetes, low MMSE score, and low BI score had a poor prognosis (P<0.05). Multivariate logistic regression showed that age ≥75 years (OR = 2.85, 95% CI: 1.63 - 4.98), disease course ≥5 years (OR = 3.12, 95% CI: 1.86 - 5.24), hypertension (OR = 2.15, 95% CI: 1.21 - 3.82), and MMSE score <15 points (OR = 4.08, 95% CI: 2.45 - 6.82) were independent factors for poor prognosis. Care strategies focus on the life care of elderly and long-term patients, conduct cognitive training for low-cognition patients, and strengthen the management of basic diseases.

**Conclusion:** Age, disease course, basic diseases, and cognitive function significantly affect the short-term prognosis of Alzheimer’s disease. Care strategies based on this can help improve patients’ short-term prognosis and improve their quality of life.

**Acknowledgements:** This work was supported by the Capacity Building and Continuing Education Center of the National Health Commission (2022YFC3602602).

## 040 | Effects of integrated nursing based on graphic communication and education mode on pulmonary function and treatment compliance of patients with COPD

Xueling Wu, Xiaoyun Zhang, Naiting Du, Yingyue Huang, Zifeng Jiang

*Department of Respiratory and Critical Care Medicine - The Second Affiliated Hospital of Soochow University, Suzhou, China*

**Objective:** To explore the impact of comprehensive nursing based on graphic and textual communication education on lung function and treatment compliance in COPD patients.

**Methods:** A retrospective study was conducted on 150 COPD patients admitted to our hospital from July 2023 to December 2023. According to different nursing measures, they were divided into a control group and an observation group, with 75 patients in each group. The control group received routine nursing care, while the observation group received comprehensive nursing care based on graphic and textual communication education mode on the basis of the control group. The rehabilitation time, treatment compliance, family satisfaction, and lung function on the day of admission and discharge [forced vital capacity (FVC), first second forced expiratory volume (FEV1), maximum ventilation volume per minute (MVV), peak expiratory flow (PEF) levels], medical fear [Children’s Medical Fear Survey (CMFS)], and quality of life [Children’s Quality of Life Scale (Ped)] were compared between the two groups. sQL4.0).

**Results:** The observation group had a statistically significant difference (P<0.05) in terms of temperature recovery, cough, wheezing, shortness of breath, disappearance of lung rales, and shorter hospital stay compared to the control group, The total compliance rate of the observation group (96.00%) and the total satisfaction rate of family members (98.67%) were higher than those of the control group (86.67%, 88.00%, respectively), and the difference was statistically significant (P<0.05), On the day of discharge, the CMFS score of the observation group was lower than that of the control group, and the PedsQL4.0 score was higher than that of the control group, with statistical significance (P<0.05), Compared with the control group, the observation group showed an increase in FVC, FEV1, MVV, and PEF levels, with statistically significant differences (P<0.05).

**Conclusion:** The application of comprehensive nursing based on graphic and textual communication education mode in COPD patients can improve treatment compliance, promote lung function recovery, shorten rehabilitation time, alleviate medical fear, improve quality of life, and increase nursing satisfaction.

## 041 | Meta-analysis of the rapid rehabilitation surgical concept in the perioperative period of hepatic resection

Lihua Wen, Qinqin Yao

*Department of Hepatobiliary and Pancreatic Surgery, the Ninth People’s Hospital of Suzhou, Suzhou, China*

**Objective:** This study ventured to clarify whether the rapid rehabilitation surgical concept is effective in improving postoperative recovery and reducing complication rates in patients undergoing hepatectomy.

**Methods:** All research was conducted through an e-database retrieval for the period from inception to 2024, and the literature was rigorously screened according to predefined criteria. Data from 11 reports totalling 1453 participants were finally enrolled, all of which compared patient recovery after hepatectomy using an ERAS protocol with a traditional model of care. The outcome indicators were postoperative complication rate, postoperative hospitalization time, duration of first venting afterwards, and pain scores.

**Results:** Eleven papers reported postoperative complication rates.The postoperative complication rate in the FTS ensemble was remarkably below that in the conventional ensemble (OR: 3.12, 95% CI: 2.51-6.87, P<0.001), suggesting that the FTS regimen significantly reduced the risk of postoperative complications. Eleven papers reviewed the length of hospital stay in the postpartum period. The duration of postpartum length of stay in the operative hospital was remarkably sharper in the employed FTS team than in the conventional team (OR: -4.85, 95% CI: -10.95 to -2.52, P<0.001), which indicated that the FTS regimen significantly reduced the duration of the postpartum length of stay in the operative hospital. Nine publications reported the time to first postoperative venting. The time to first venting in the immediate afteroperative period was remarkably shorter in the adopting FTS team than in the conventional team, (OR: 3.52, 95% CI: 2.18-6.15, P<0.001), which indicated FTS regimen could significantly shorten the time to first venting in the immediate afteroperative period. Six papers reported pain scores. The use of FTS resulted in postsurgical pain ratings that showed markedly fewer in the FTS employed team than in the conventional team (OR: -0.65, 95% CI: -0.91 to -0.46, P<0.001), which suggests that FTS regimen significantly reduces the postoperative pain ratings.

**Conclusion:** The current research demonstrates that the rapid rehabilitation surgical philosophy applied to hepatic hepatectomy in the perioperative period effectively promotes the postoperative rehabilitation process, reduces complications, and improves the overall satisfaction of the patients.

## 042 | Application effect of traditional Chinese medicine nursing combined with external application intervention in elderly patients with atopic dermatitis

Jing Wu, Lu Ge

*Department of Geriatrics, the Third Affiliated Hospital of Zhejiang Chinese Medicine University, Hangzhou, China*

**Objective:** to explore the effect of traditional Chinese medicine nursing combined with external application intervention on the clinical efficacy of elderly atopic dermatitis patients.

**Methods:** 120 cases of elderly atopic dermatitis patients in our hospital were selected from June 2023 children in June 2024 and randomly divided into control group and observation group, 60 cases each. The control group used conventional care, and the observation group added Chinese medicine care combined with external application intervention on the basis of conventional care. Comparison of the two groups of patients with the area of skin lesions, the degree of itching, serum IgE levels, quality of life scores, anxiety and depression scores and nursing satisfaction.

**Results:** Clinical indicators: lesion area, itchiness, and IgE level were lower than those before nursing care in both groups (P<0.05), and lesion area, itchiness, and IgE level were better than those in the control group after nursing care in the observation group (P<0.05). Quality of life: the DLQI scores of patients in both groups were better than those before care (P<0.05), and the observation group was better than the control group after care (P<0.05). Psychological assessment: SDS and SAS were lower than before care in both groups (P<0.05), and lower in the observation group than in the control group (P<0.05). Therapeutic efficacy: the therapeutic efficacy of 93.33% in the observation group was higher than the therapeutic efficacy of 70.00% in the control group (P<0.05). Nursing satisfaction: the total satisfaction rate of 98.33% in the observation group was higher than 93.33% in the control group (P<0.05).

**Conclusion:** Chinese medicine nursing care combined with external application intervention can not only effectively improve the clinical symptoms and psychological status of elderly atopic dermatitis patients, but also enhance their quality of life, which is an effective nursing approach worth promoting. Follow-up studies can further explore more personalized and precise TCM nursing programs to enhance the efficacy and meet the special needs of elderly patients.

## 043 | Differences in spinal sagittal morphology between adolescent idiopathic scoliosis patients and healthy adolescents: a cross-sectional study

Gen Zhang^1^, Jing Lu^2^, Jun Yu^3^

^*1*^*Department of Traditional Chinese Orthopedics, Wuxi Ninth People’s Hospital Affiliated to Soochow University, Wuxi, China;* ^*2*^*Wuxi National Fitness Monitoring Center, Wuxi, China;* ^*3*^*Rehabilitation Centre, Wuxi Ninth People’s Hospital Affiliated to Soochow University, Wuxi, China*

**Objective:** Adolescent idiopathic scoliosis (AIS), the most common spinal deformity in adolescents, is a three-dimensional deformity of the coronal, sagittal, and horizontal surfaces of the spine. Some studies have suggested that coronal malformations in AIS patients can lead to changes in sagittal plane parameters. With the research on spinal deformities and spinal surgery biomechanics in recent years, scholars have gradually realized the importance of sagittal balance. Biomechanical research shows that the human spine always maintains a sagittal dynamic balance.

**Methods:** At present, most domestic and foreign studies focus on the interaction between different segments of the spinal sagittal plane in AIS patients. Most studies lack comparison with healthy adolescents. Therefore, the purpose of this study was to conduct research on the spinal sagittal surface morphology of AIS patients and healthy adolescents and analyze the causes of its changes and its clinical significance.

A retrospective study of 100 AIS patients and 66 age- and gender-matched healthy adolescents admitted to Wuxi No. 9 Hospital Affiliated to Soochow University from January 2018 to May 2023 was conducted. All subjects received full-length anterior and lateral spinal radiographs. Sagittal plane parameters were measured independently by two researchers: (1) Cervical Saginal Aligllment (CSA), (2) Lower Cervical Lordosis Angle, (3) Thoracic kyphosis (TK), (4) Lumbar Lordosis (LL), (5) Sacral Slop (SS), (6) Sagittal Vertical Axis (SVA). The statistical difference in sagittal plane parameters between AIS patients and healthy adolescents was compared using an independent sample T-test.

**Results:** Compared with healthy adolescents, there were no significant statistical differences in the Lower Cervical Lordosis Angle and SS size in AIS patients (*P*>0.05). AIS patients had larger CSA (t=4.400, *P*<0.001), smaller TK (t=2.793, *P* =0.006), larger LL (t=-7.616, P<0.001) and larger SVA (t=2.233, *P* =0.027).

**Conclusion:** CSA, TK, LL and SVA are important indexes to judge AIS sagittal plane imbalance. In the AIS correction training, attention should be paid to the integration of the neck, thoracic and lumbar vertebrae. The most important thing is to conduct healthy education to prevent scoliosis and get thorough improvement from the cultivation of habits.

## 044 | Efficacy of Combined Home Balloon Training and Biofeedback Therapy on Pelvic Floor Relaxation-Type Constipation

Lei Tong^1^, Hezhai Yin^1^, Qi Wang^1^, Mengting Qin^1^, Zhenya Zhu^2^

^*1*^*Anorectal, Jiaxing Hospital of Traditional Chinese Medicine, Jiaxing, China;* ^*2*^*Orthopedics andTraumatology, Jiaxing Hospital of Traditional Chinese Medicine, Jiaxing, China*

**Objective:** To investigate the therapeutic effect of home balloon training combined with biofeedback therapy on pelvic floor relaxation-type constipation and its impact on anorectal dynamics, constipation status, and other relevant parameters.

**Methods:** A total of 106 patients with pelvic floor relaxation-type constipation treated at our hospital were randomly divided into two groups: a conventional group and a combined group. The conventional group received biofeedback therapy, while the combined group underwent additional home balloon training. The two groups were compared in terms of overall treatment efficacy, severity of constipation symptoms, anorectal dynamics, gastrointestinal hormone levels, quality of life, and adverse reactions.

**Results:** The total effective rate in the combined group (88.68%) was significantly higher than that in the conventional group (71.70%) (P <0.05). Levels of motilin (MTL), anal resting pressure, substance P (SP), and anal contraction pressure were significantly higher in the combined group compared to the conventional group (P <0.05). In contrast, Cleveland Constipation Score (CCS) and rectal defecation threshold were significantly lower in the combined group (P <0.05). PAC-QOL (Patient Assessment of Constipation Quality of Life) scores were also significantly lower in the combined group compared to the conventional group (P <0.05). There was no significant difference in the incidence of adverse reactions, such as allergies or pain, between the two groups (P>0.05).

**Conclusion:** Home balloon training combined with biofeedback therapy effectively alleviates constipation symptoms and improves anorectal dynamics in patients with pelvic floor relaxation-type constipation. This combined treatment demonstrates ideal therapeutic outcomes and holds significant clinical application value.

## 045 | The effect of chronic insomnia on cognitive decline in patients with AD

Lan Zou^1^, Qi Wang^1^, Han Mei^2^, Zehua Yang^3^

^*1*^*China Construction Third Engineering Bureau Wuhan Central Hospital, Wuhan, China;* ^*2*^*Clinical Research Unit of Changsha Economic Development Zone Hospital, Changsha, China;* ^*3*^*Department of Drug Inspection, Hunan Drug Inspection Center, Changsha, China*

**Objective:** To explore the impact of chronic insomnia on cognitive decline in patients with Alzheimer’s disease (AD).

**Methods:** A total of 97 AD patients from June 2023 to August 2024 were selected as the subjects. The sleep quality of the patients was evaluated according to the Pittsburgh Sleep Quality Inventory (PSQI), and the patients were divided into the chronic insomnia group and the non-chronic insomnia group in combination with the diagnostic criteria of chronic insomnia. The cognitive function status of the two groups was evaluated by using the Chinese version of the Mini-Mental State Screening Scale (CMMSR). The functional independent use function was evaluated by using the Functional Independence Rating Scale (FIM), The degree of cognitive decline in patients was evaluated using the Global Decline Scale (GDS), and the cognitive decline of the two groups of AD patients was compared. The Pearson correlation analysis software was used to conduct a correlation analysis between the PSQI score of AD patients and cognitive decline.

**Results:** Among 97 AD patients, 32 cases (accounting for 32.99%) were diagnosed with chronic insomnia by relevant criteria. The PSQI score of the chronic insomnia group was higher than that of the non-chronic insomnia group (P<0.05). The time/place orientation, food naming ability, immediate memory, attention and calculation ability, delayed memory, graphic replication and language ability in the chronic insomnia group were lower than those in the non-chronic insomnia group (P<0.05). The scores of self-care, sphincter control, transfer, walking, movement and cognition in the FIM scale of the chronic insomnia group were lower than those of the non-chronic insomnia group (P<0.05). The GDS was higher than that of the non-chronic insomnia group (P<0.05). The Pearson correlation results indicated that the PSQI score of AD patients was positively correlated with the CMMSR and FIM scores (P<0.05), It was negatively correlated with GDS (P<0.05).

**Conclusion:** The proportion of chronic insomnia in AD patients is relatively high. These patients are accompanied by varying degrees of cognitive decline, resulting in functional decline and reduced functional independence of the patients. Moreover, there is a correlation between chronic insomnia and cognitive decline. Actively taking effective measures to improve the sleep quality of patients is helpful to delay cognitive decline.

## 046 | Construction of intelligent decision-making model for priority ranking of exercise intervention in elderly patients with chronic disease comorbidity

Loucheng Yu, Haidong Xu

*College of Physical Education, Changzhou University, Changzhou, China*

**Objective:** With the acceleration of population aging, the phenomenon of chronic diseases in the elderly is becoming more and more common, which seriously affects the quality of life and health of patients. Exercise intervention is an important means to improve the health status of elderly patients with chronic diseases, and the scientific rationality of its priority ranking is very important. In this study, the key elements are determined by the comprehensive application of multidisciplinary theories such as exercise physiology and geriatrics, and the intelligent decision-making model is constructed by means of data analysis method and artificial intelligence algorithm. The model mainly finds that different pathological characteristics of chronic diseases, individual differences of patients, and safety and effectiveness indicators of exercise intervention have different weights in priority ranking. The model can provide accurate exercise intervention guidance for elderly patients with chronic diseases, which is helpful to optimize the exercise intervention program and improve the effect of exercise intervention. It is of great significance to improve the health status of elderly patients with chronic diseases.

**Methods:** The purpose of this study is to construct an intelligent decision-making model to scientifically and reasonably determine the priority of exercise intervention for elderly patients with chronic diseases. By comprehensively analyzing the key factors such as the pathological characteristics of chronic diseases, individual differences of patients and the safety and effectiveness indicators of exercise intervention, a model that can accurately evaluate the suitability of different exercise intervention methods for elderly patients with chronic diseases was constructed by using data analysis methods and artificial intelligence algorithms. The model will provide decision support for clinicians, rehabilitation practitioners and patients themselves, and help them to quickly and accurately determine the optimal exercise intervention priority ranking according to the specific conditions of patients in many exercise intervention options, so as to realize personalized and accurate exercise intervention, and finally improve the health status and quality of life of elderly patients with chronic diseases.

**Results:** The intelligent decision-making model can integrate the key factors such as the pathological characteristics of chronic diseases, individual differences, and the safety and effectiveness indicators of exercise intervention in elderly patients with chronic diseases. Through the comprehensive analysis and algorithm calculation of these elements, each patient is tailored. Customize the exercise intervention program to provide more scientific and accurate guidance.

**Conclusion:** The purpose of this study is to construct an intelligent decision-making model for priority ranking of exercise intervention in elderly patients with chronic diseases. The construction process was determined by key elements, including the analysis of pathological characteristics of chronic diseases, individual differences of patients, and the identification of safety and effectiveness indicators of exercise intervention. The data analysis method is used to process the key element data, and the appropriate artificial intelligence algorithm is selected for integration and weight distribution, and finally the intelligent decision model is formed.

**Acknowledgements:** Ministry of Education Humanities and Social Sciences Fund Project: Research on the Intelligent Model of Exercise Intervention for Elderly Chronic Disease Groups (Project No.: 21YJA890039).

## 047 | Effect of Family Participation in Companionship Plan Nursing on The Quality of Life of Adolescent Depression Patients

Lei Gong, Chunqi Ai, Shun Zeng, Jun Xu, Hongmei Chen, Xiong Chen

*Mental Health Center, Taihe Hospital, Shiyan, China*

**Objective:** This study aimed to assess the impact of implementing a family participation in companionship plan nursing on the quality of life of adolescent depression sufferers.

**Methods:** Sixty-eight adolescent depression sufferers were selected from our hospital between March 2022 and March 2023 and randomly assigned to two groups. The control group (n=34) received routine nursing care, while the observation group (n=34) underwent family participation in companionship plan nursing. Various scales and questionnaires, including HAMD, MADS, CSQ, SSRS, BMQ, ITAQ, ASWS, BSC-CV, GQOLI-74, and nursing satisfaction, were utilized for comparison.

**Results:** After 12 weeks of intervention, the observation group exhibited lower HAMD and MADS scores, negative coping and medication anxiety scores, along with higher positive coping, social support, perceived medication necessity, and insight and treatment attitude scores compared to the control group (all P<0.05). Additionally, the observation group showed improvements in Adolescent Sleep-Wake Scale scores, various dimensions of GQOLI-74, and nursing satisfaction, while demonstrating a lower Beck Suicide Intention Scale score (P<0.05).

**Conclusion:** Implementing family participation in companionship plan nursing proved effective in reducing depression severity, fostering positive coping strategies, enhancing social support, improving medication beliefs, insight, treatment attitudes, sleep quality, and reducing suicidal tendencies among adolescent depression sufferers. This approach holds promise for enhancing overall life quality in this demographic.

## 048 | Application Effect of Intervention Model Based on Early Warning Scoring Model in Elderly Patients with Myocardial Infarction and Its Impact on Social Support level

Xiaoyan Hu^1^, Bairu Xiong^1^, Lei Xiang^1^, Biao Wang^1^, Yang Liu^1^, Xianfeng Wen^1^, Xuezhu Liao^1^, Luhua Chen^2^

^*1*^*Department of Cardiology, Jingzhou Second People’s Hospital, Jingzhou, China;* ^*2*^*Department of Cardiovascular Medicine, Tongji Hospital, Tongji Medical College, Hua Zhong University of Science and Technology, Wuhan, China*

**Objective:** To explore the application effect of the intervention model based on the early warning scoring model in elderly patients with myocardial infarction and its effect on the level of social support.

**Methods:** A total of 94 elderly patients with myocardial infarction from June 2022 to August 2024 were selected as the subjects and divided into two groups by the envelope method, with 47 cases in each group. The control group received conventional care, and the observation group received the intervention model based on the early warning scoring model. Both groups completed 4 weeks of intervention. The self-management ability, social support level and satisfaction of the two groups were compared.

**Results:** After the intervention, the self-management ability of the two groups was improved. The scores of emotional cognition, treatment compliance, disease knowledge acquisition, first aid management, symptom management, bad habit management and general life management in the observation group were higher than those in the control group (*P*<0.05). The social support level of the two groups was improved after the intervention. The objective, subjective support and social support utilization scores of the observation group were higher than those of the control group (*P*<0.05). The satisfaction of the observation group after the intervention was higher than that of the control group (*P*<0.05).

**Conclusion:** The intervention model based on the early warning scoring model has a good effect on elderly patients with myocardial infarction, can improve patients’ self-management ability, improve the level of social support, and obtain higher satisfaction, which is worthy of promotion and application.

## 049 | A study on improving the diabetes remission rate in overweight/obese patients with type 2 diabetes through triple therapy of short-term insulin intensification, dapagliflozin and metformin

Hao Hu, Wanwan Zhang, Wenwen Yin

*Department of Endocrinology, The Affiliated Xuzhou Municipal Hospital of Xuzhou Medical University, Xuzhou, China*

**Objective:** To explore the research value of short-term insulin intensification, dapagliflozin and metformin triple therapy in improving the diabetes remission rate in overweight/obese patients with type 2 diabetes.

**Methods:** A total of 148 overweight/obese patients with type 2 diabetes mellitus (T2DM) in our hospital were randomly divided into the experimental group and the control group, with 74 cases in each group. The control group received short-term intensive insulin treatment, while the experimental group was additionally combined with dapagliflozin and metformin on this basis. The waist circumference, body mass index (BMI), systolic blood pressure, diastolic blood pressure, glucose metabolism indicators [fasting blood glucose (FBG), fasting insulin (FINS), glycated hemoglobin (HbAlc), insulin resistance index HOMA-IR)], abdominal subcutaneous fat area, abdominal visceral fat area, occurrence of adverse reactions, and diabetes remission rate were compared between the two groups.

**Results:** Compared with before treatment and after 12 weeks of treatment in the control group, the waist circumference of the experimental group significantly decreased after 12 weeks of treatment (P<0.05). Compared with before treatment and after 12 weeks of treatment in the control group, the levels of FBG, HbA1c, FINS and HOMA-IR in the experimental group were significantly decreased after 12 weeks of treatment (P<0.05). Compared with before treatment and after 12 weeks of treatment in the control group, the area of abdominal subcutaneous fat and the area of abdominal visceral fat in the experimental group significantly decreased after 12 weeks of treatment (P<0.05). There was no significant difference in the incidence of adverse reactions between the two groups (P>0.05). The remission rate of diabetes in the experimental group was significantly higher than that in the control group (P<0.05).

**Conclusion:** The triple therapy of short-term insulin intensification, dapagliflozin and metformin in the treatment of overweight/obese patients with type 2 diabetes has a certain clinical efficacy. It can improve blood glucose levels, reduce visceral fat area, and has high safety. It is conducive to improving the remission rate of diabetes and should be given attention.

## 050 | Genetics of Sports Injuries and Personalized Treatment Strategies: A Study on ACL Injury Risk Assessment and Intervention in Soccer Players Based on Genetic Polymorphism

Mengmeng Wang^1^, Yangwen He^2^, Dehua Liu^1^, Somi Lee^3^

^*1*^*Sports Department, Jiangsu University of Technology, Changzhou, China;* ^*2*^*Department of Orthopedics, West Taihu Hospital, Changzhou, China;* ^*3*^*Graduate School Sports Department, Sangmyung University, Seoul, Korea*

**Objective:** Anterior cruciate ligament (ACL) injuries are prevalent and debilitating conditions that significantly affect athletes, especially in high-impact sports such as soccer. Recent advancements in genetics have paved the way for exploring the genetic underpinnings of sports injuries, offering insights into individual susceptibility and paving the path towards personalized treatment strategies. This study aims to assess the ACL injury risk in soccer players based on genetic polymorphism and to develop tailored intervention strategies to mitigate this risk.

**Methods:** A cohort of professional soccer players was analyzed for specific genetic markers associated with an increased risk of ACL injuries. The study employed a case-control design, wherein soccer players with a history of ACL injuries were compared to those without such injuries. Genomic DNA was extracted from participants’ blood samples, and single nucleotide polymorphisms (SNPs) known to influence ACL injury risk were genotyped. Based on the genotyping results, personalized intervention strategies, including targeted physical therapy programs and preventive measures, were designed and implemented. The effectiveness of these interventions was evaluated through follow-up assessments and injury occurrence monitoring.

**Results:** The genotyping analysis identified several SNPs significantly associated with ACL injury risk among the soccer players studied. Players carrying these risk alleles were found to have a higher incidence of ACL injuries compared to those without. The personalized intervention strategies, tailored according to the genetic risk profiles, demonstrated a significant reduction in the occurrence of ACL injuries in the high-risk group. Participants also reported improved performance and decreased injury-related downtime.

**Conclusion:** This study underscores the potential of genetic polymorphism in assessing ACL injury risk among soccer players and highlights the efficacy of personalized intervention strategies in mitigating this risk. By integrating genetic information into the development of injury prevention programs, this research contributes to the evolving field of personalized medicine in sports, offering a novel approach to injury prevention and athlete care. Further research is encouraged to explore the broader applicability of genetic-based risk assessment and personalized interventions across different sports and injury types.

## 051 | Effect of transurethral resection of bladder tumor combined with gemcitabine infusion chemotherapy on postoperative recurrence of bladder cancer

Kang Li^1^, Lingshan Huang^2^, Dan Yuan^1^, Yu Wang^1^, Mingsong Wang^1^, Liang He^3^

^*1*^*Department of Urology, 363 Hospital, Chengdu, China;* ^*2*^*Medical Department, 363 Hospital, Chengdu, China;* ^*3*^*Oncology Department, 363 Hospital, Chengdu, China*

**Objective:** The objective of this study was to evaluate the effect of transurethral resection of bladder tumor combined with gemcitabine infusion chemotherapy on the postoperative recurrence of bladder cancer. In this study, 204 patients with bladder cancer were included and divided into observation group and control group. The observation group received transurethral resection of bladder tumor followed by gemcitabine infusion chemotherapy, and the control group received transurethral resection of bladder tumor. Long-term follow-up was conducted to observe the efficacy and postoperative recurrence of patients in both groups to evaluate the effect of combined therapy.

**Methods:** A total of 204 patients with bladder cancer participated in this study. The observation group included 102 patients who received gemcitabine infusion chemotherapy after transurethral resection of bladder tumor. The control group included 102 patients who underwent simple transurethral resection of bladder tumors. The follow-up period was 5 years. Assessment indicators such as urine cytology, cystoscopy and imaging were monitored regularly, and the curative effect and postoperative recurrence were recorded.

**Results:** In the observation group, 88 patients had no recurrence during the postoperative follow-up, and the recurrence rate was 13.7%. In the control group, 65 patients had postoperative recurrence, with a recurrence rate of 33.3%. After statistical analysis, the recurrence rate of observation group was significantly lower than that of control group (P&lt, 0.05). In addition, the observation group had significantly higher overall survival and tumor-free survival than the control group (P&lt, 0.05).

**Conclusion:** Transurethral resection of bladder tumor combined with gemcitabine infusion chemotherapy is effective in the treatment of bladder cancer. The results of this study indicate that combination therapy can significantly reduce the recurrence rate of bladder cancer after surgery and improve overall survival and tumor-free survival. Therefore, transurethral resection of bladder tumor combined with gemcitabine infusion chemotherapy should be considered as an important treatment option for patients with bladder cancer. This study provides useful guidance and enlightenment for clinical practice, which is helpful to improve the treatment effect and quality of life of patients with bladder cancer.

## 052 | Topological Insights into Community Structure and Resource Allocation in Medical Networks

Meili Zhang^1^, Weili Liu^1^, Yue Yang^1^, Xuanhang Feng^2^, Hongji Zhang^2^

^*1*^*Department of the foundation, Dalian Naval Academy, Dalian, China;* ^*2*^*Cadet team, Dalian Naval Academy, Dalian, China*

**Objective:** This study aims to explore the application of topology in the detection of communities within medical networks, specifically focusing on the effectiveness of a topology-based potential community detection algorithm in medical network analysis. The goal of the research is to evaluate the accuracy of the algorithm in identifying community structures within medical networks and analyze its potential impact on the allocation of medical resources and patient management.

**Methods:** This study employs a topology-based potential community detection algorithm that combines label propagation and Topological Data Analysis (TDA). We initially represent entities in the medical network, such as patients, doctors, and medical equipment, as nodes in the network, and relationships between them, such as referral relationships and referral paths, as edges. By constructing Vietoris-Rips complexes (VR complexes), we transform these network data into topological spaces to extract key topological features. Using persistent homology theory, we identify holes and voids in the network, features that help reveal community structures within the network. We selected datasets containing medical network data, including intra-hospital referral networks and patient interaction networks, to train and test our model.

**Results:** The experimental results indicate that the topology-based potential community detection algorithm performs well in the detection of communities within medical networks. In the analysis of intra-hospital referral networks, the algorithm successfully identified community structures centered around specific departments, which highly correspond to the actual departmental distribution in hospitals. In patient interaction networks, the algorithm revealed social network structures among patient groups, which are related to common disease types and treatment needs. Specific data showed that the algorithm achieved an average modularity ($Q$) score of 0.76, 10% higher than traditional algorithms, indicating tighter connections within communities. Moreover, the algorithm also demonstrated high accuracy in identifying key nodes, such as expert doctors and influential patients, which is crucial for optimizing the allocation of medical resources and improving patient satisfaction.

**Conclusion:** This study concludes that topology has significant application value in the detection of communities within medical networks. The topology-based potential community detection algorithm can effectively identify community structures in medical networks, providing a new perspective for the optimization of medical resource allocation and patient management. The application of this algorithm is expected to improve the efficiency and quality of medical services and promote the development of personalized medicine. Future research can further explore the application of this algorithm in different types of medical networks and combine it with other data science methods to achieve a more comprehensive analysis of medical networks.

**Acknowledgements:** This research was financially supported by the scientific research and development fund project of Dalian Naval Academy.

## 053 | Evaluation of the effect of clinical biochemistry test technology course in superstar learning platform by big data

Hongxia Gao^1^, Xinhui Ma^2^, Guoqing Wang^1^

^*1*^*BeihuaUniversity, College of Medical Technology, Jilin, China;* ^*2*^*Heilongjiang Provincial General Hospital of the People ‘s Armed Police Force of China, Heilongjiang, China*

**Objective:** The study of big data in evaluating the effectiveness of clinical biochemistry testing technology courses on the Chaoxing learning platform has important theoretical and practical significance. From a theoretical perspective, it helps to enrich and improve the theoretical system for evaluating the effectiveness of online education courses, and explore new paths and methods for the application of big data in the field of medical education. In practice, through big data evaluation, it is possible to accurately identify the problems that exist in the teaching process of the course, such as whether the difficulty level of the teaching content is moderate, whether the teaching methods are effective, etc., and provide targeted suggestions for improving the quality of the course. At the same time, optimizing teaching modes, improving students’ learning outcomes and satisfaction, cultivating professional talents that better meet clinical needs, and promoting the development of medical education

**Methods:** To build a big data evaluation model, the first step is to select indicators reasonably. Based on the collected student learning behavior data, indicators such as video viewing duration, homework submission status, test scores, and interaction frequency can be selected. The video viewing duration indicator can reflect students’ learning engagement, homework submission reflects their learning attitude and knowledge application ability, test scores are directly related to knowledge mastery, and interaction frequency represents learning participation. After determining the indicators, it is necessary to scientifically and reasonably determine the weights of each indicator. Scientific methods such as Analytic Hierarchy Process can be used, combined with the experience of education experts and the characteristics of the curriculum, to quantitatively evaluate the importance of various indicators and determine their weights. For courses such as clinical biochemistry testing technology that closely integrate theory with practice, the weight of test scores and homework submission indicators can be appropriately increased to highlight the importance placed on students’ knowledge application and practical abilities. The evaluation model constructed through rigorous indicator selection and weight determination process has high scientific and feasibility, and can comprehensively and accurately evaluate the effectiveness of the course, providing strong data support for course optimization.

**Results:** This study evaluates the effectiveness of clinical biochemistry testing technology courses on the Chaoxing learning platform based on big data and has made a series of important findings. Collecting student learning behavior data through the Chaoxing learning platform, covering various aspects such as video viewing duration, homework submission status, and discussion forum speeches, these data reflect the course effectiveness from different perspectives. For example, the duration of learning can reflect students’ level of investment in knowledge, test scores intuitively demonstrate their level of knowledge mastery, and the frequency of interaction reflects the interactivity and attractiveness of the course. The big data evaluation model constructed based on this has high scientificity and feasibility by scientifically selecting indicators and reasonably determining weights, and can comprehensively and objectively evaluate the effectiveness of courses.

**Conclusion:** Big data has played a crucial role in course evaluation, breaking the limitations of traditional assessment methods. It provides rich data dimensions, allowing assessment to no longer be limited to a single exam score or homework grading, but to consider multiple aspects of students’ learning process, achieving comprehensive evaluation. At the same time, big data evaluation is real-time, and teachers can adjust teaching strategies in a timely manner based on real-time feedback data to improve teaching effectiveness. Moreover, the evaluation based on big data is relatively objective, reducing the interference of subjective human factors and making the evaluation results fairer and more accurate.

## 054 | Neurocognitive load assessment and adaptive interactive optimization for long-term driving

Julin Wen, Xueyan Wen

*Ganzhou polytechnic, Ganzhou, China*

**Objective:** Long term driving scenarios are widely present in modern transportation systems, such as long-distance freight transportation and self-driving tours. However, prolonged driving can easily lead to excessive cognitive load on the driver’s nerves, which in turn can cause problems such as fatigue driving and distraction, seriously threatening driving safety. The aim of this study is to construct an accurate long-term driving neurocognitive load assessment system, and based on this, design effective adaptive interactive optimization strategies to enhance the safety of long-term driving. In terms of evaluating technical means, a multimodal neurocognitive load assessment system is constructed by comprehensively using methods such as EEG signal analysis, eye tracking, and physiological signal monitoring, fully leveraging the advantages of each method and compensating for the limitations of a single method. In terms of adaptive interaction optimization strategy, starting from the optimization of information presentation mode, interaction timing, and personalized adaptive interaction scheme customization, based on the results of neurocognitive load assessment, adjust the information presentation mode of the in vehicle information system, determine the optimal interaction timing, and consider individual differences to customize personalized interaction schemes. Through experimental verification, the neurocognitive load assessment method in this study is effective, and the adaptive interactive optimization strategy can significantly reduce the cognitive load of drivers, improve long-term driving safety, and provide new ideas and methods for ensuring long-term driving safety.

**Methods:** The purpose of this experiment is to verify the effectiveness of the constructed long-term driving neurocognitive load assessment method, as well as the positive effect of the adaptive interactive optimization strategy based on neurocognitive load on improving driving safety. The selection of experimental subjects takes into account factors such as age and driving experience to ensure a certain level of representativeness. The experimental setting simulates a real long-term driving environment, including highways, urban roads, and mountainous roads, to comprehensively examine the changes in neurocognitive load under different driving conditions. In terms of experimental equipment, high-precision EEG signal acquisition devices are used to record the driver’s EEG activity in real time, Using advanced eye tracking devices to accurately capture eye movement data, simultaneously using physiological signal monitoring instruments to obtain physiological signals such as heart rate and skin conductance. The evaluation indicators selected parameters closely related to neurocognitive load, such as specific band power in EEG signals, gaze time and scanning path length in eye movement indicators, and heart rate variability in physiological signals.

**Results:** Conduct in-depth analysis of the collected experimental data using statistical methods. Firstly, regarding the neurocognitive load assessment method, the effectiveness is verified by comparing the changing trends of different assessment indicators under different driving durations and road conditions. For example, analyzing the changes in band power reflecting cognitive load in EEG signals with increasing driving time, as well as the differences in eye movement indicators and physiological signals under different road conditions, in order to determine that the constructed multimodal evaluation system can accurately reflect the driver’s neurocognitive load status. Secondly, for the adaptive interaction optimization strategy, the cognitive load evaluation indicators of drivers before and after adopting the optimization strategy are analyzed Driving behavior data to verify its role in reducing cognitive load and improving driving safety. If the experimental results show that after adopting the optimization strategy, the cognitive load index of the driver is significantly reduced, and the driving behavior is more stable, such as reduced vehicle speed fluctuations and lane departure frequency, it can be proved that the adaptive interactive optimization strategy has a positive effect.

**Conclusion:** This study focuses on the neurocognitive load assessment and adaptive interactive optimization of long-term driving, and has achieved a series of important results. In terms of neurocognitive load assessment methods, in-depth exploration was conducted on techniques such as EEG signal analysis, eye tracking, and physiological signal monitoring, and a multimodal comprehensive evaluation system was constructed. EEG signal analysis, with its high temporal resolution, can accurately capture subtle changes in neurocognitive activity, Eye tracking provides a non-invasive way to visually reflect the correlation between driver attention allocation and cognitive load, Physiological signal monitoring provides important basis for cognitive load assessment based on real-time and objectivity. By comprehensively applying these methods, a comprehensive and accurate assessment of long-term driving neurocognitive load has been achieved

## 055 | The efficiency of digital design-driven portable medical equipment for primary clinical diagnosis is improved

Xiongwei Jia

*School of Mechanical and Electrical Engineering, Xi’an Traffic Engineering University, Xi’an, China*

**Objective:** With the continuous penetration of digital technology in the medical field, the design and development of medical equipment are undergoing profound changes. As the cornerstone of the healthcare system, primary healthcare faces an urgent need to improve diagnostic efficiency. Portable medical devices driven by digital design have emerged, integrating advanced technologies such as digital signal processing and embedded systems, with features such as precise sensor technology, fast detection and result feedback, and integrated multiple detection functions. This type of equipment plays an important role in improving the accuracy and efficiency of primary clinical diagnosis, as well as expanding the scope of diagnosis. The research conclusion indicates that portable medical devices driven by digital design are expected to significantly improve the efficiency of primary clinical diagnosis, but at the same time, they face challenges in equipment cost, personnel adaptability, and data security and privacy protection. Cost control strategies, personnel training programs, and data security measures need to be implemented to better promote the development of primary healthcare.

**Methods:** This study aims to analyze in depth the role of digital design driven portable medical devices in enhancing the diagnostic efficiency of primary clinical practice. Through research on improving diagnostic accuracy, enhancing diagnostic efficiency, and expanding diagnostic scope, comprehensively evaluate its positive impact on grassroots clinical diagnosis. At the same time, practical and feasible development suggestions are proposed to address the potential problems that the device may face in grassroots applications, providing theoretical basis and practical guidance for promoting the widespread application of digital design driven portable medical devices in grassroots settings

**Results:** This study deeply analyzed the role of digital design driven portable medical devices in improving the diagnostic efficiency of grassroots clinical practice. Research has found that with the help of digital design, portable medical devices have demonstrated significant advantages in primary clinical diagnosis. By using precise sensor technology to obtain accurate physiological parameters and applying data analysis and optimization algorithms to deeply explore the detection data, the diagnostic accuracy has been greatly improved. At the same time, rapid detection and result feedback, as well as simplified operation processes, effectively improve diagnostic efficiency and save a lot of time for grassroots medical staff. In addition, integrating multiple detection functions and remote diagnostic support further expands the scope of diagnosis, enabling grassroots medical institutions to carry out more detection projects and obtain expert resource support. These achievements fully demonstrate the important value of digital design driven portable medical devices in improving the efficiency of primary clinical diagnosis, and are of great significance for improving the quality of primary medical services and alleviating the uneven distribution of medical resources.

**Conclusion:** With the rapid development of artificial intelligence technology, its deep integration with portable medical devices will be an important research direction. Artificial intelligence technologies such as machine learning and deep learning are expected to further enhance the diagnostic efficiency of devices, achieving more intelligent and accurate diagnosis. On the other hand, the trend of device miniaturization and multifunctional integration is worth paying attention to. In the future, it is necessary to continuously explore how to integrate more detection functions in smaller device volumes to meet the growing diagnostic needs at the grassroots level. In addition, building a digital ecosystem for grassroots healthcare and achieving effective integration and collaborative diagnosis of portable medical devices, medical information platforms, and other resources is also a key direction for future research. Through in-depth research on these directions, it is expected to provide stronger technical support for the development of primary healthcare and promote the comprehensive improvement of primary healthcare service levels.

**Acknowledgements:** 2022 Phase Research Results of the “Mechanical Design” Teaching Team of Xi’an Traffic Engineering University.

## 056 | The key technology and research of automatic production of medical machinery manufacturing under intelligent manufacturing mode

Xueping Li

*Nanchang Hangkong University, Nanchang, China*

**Objective:** Under the background of the transformation of global manufacturing industry to intelligence, intelligent manufacturing has become the core force to promote the development of various industries, medical machine. The machinery manufacturing industry is no exception. In order to improve the quality and efficiency of medical machinery manufacturing, to meet the growing market demand, the. The research on the key technology of dynamic production is imminent. Through literature research, current situation analysis and case study, this paper makes an in-depth analysis. The key technologies of automatic production of medical machinery manufacturing under intelligent manufacturing mode are analyzed. The study found that advanced sensor technology, precise control.In the intelligent manufacturing mode, it is urgent to study the key technologies of automatic production of medical machinery manufacturing. At present, the medical machinery manufacturing industry is facing increasingly fierce market competition and rising market demand. Traditional production methods have been difficult to meet the needs of industry development. Therefore, in-depth study of the key technologies of automated production will help break through the existing production bottlenecks and enhance the core competitiveness of enterprises. At the same time, it is of great value to promote the intelligent transformation of the medical machinery manufacturing industry, which can help the industry achieve high-quality development, provide patients with better and more advanced medical device products, and then promote the development of the entire medical industry.

**Methods:** In terms of technical problems, we should strengthen technical research and development cooperation, and carry out industry-university-research cooperation among enterprises, scientific research institutions and universities to jointly overcome key technical problems such as data compatibility and system integration, and share research and development results. For the cost problem, enterprises should optimize cost management, make full cost-benefit analysis before equipment purchase, and make reasonable planning. Equipment configuration, at the same time, through technological innovation and process improvement, production costs are reduced and resource utilization efficiency is improved.

**Results:** In the intelligent manufacturing mode, the automatic production of medical machinery manufacturing involves a variety of key technologies. With its data acquisition and processing principle, advanced sensor technology realizes real-time monitoring and adaptive adjustment of medical machinery manufacturing process, and significantly improves manufacturing accuracy and quality. Precision control technology, including open-loop and closed-loop control, can effectively improve production efficiency and quality by optimizing production process and ensuring product consistency. The application of high-efficiency industrial robot technology in medical machinery assembly, welding, detection and other links brings the advantages of improving production automation, reducing labor intensity and improving production flexibility.

**Conclusion:** In the future, the research on automatic production of medical machinery manufacturing under intelligent manufacturing mode can further deepen the key technologies. For example, for advanced sensor technology, more accurate and efficient data acquisition and processing methods can be explored, for precision control technology, more intelligent and adaptive control algorithms are studied. Develop a more flexible and intelligent robot system for industrial robot technology. At the same time, expanding the application field is also an important direction. These key technologies can be applied to more types of medical machinery manufacturing scenarios to further promote the intelligent transformation of the medical machinery manufacturing industry to meet the growing market demand and improve product quality requirements.

## 057 | Research progress of sustained and controlled release preparation technology and its application in oral drugs

Lyu Jie^1^, Dengke Long^2^

^*1*^*College of Pharmaceutical and Environmental Engineering, Chongqing Industry and Information Vocational College, Chongqing, China;* ^*2*^*Chongqing Development Center for Responding to Climate Change (Chongqing Resources and Environment Trading Center), Chongqing, China*

**Objective:** The purpose of this study is to comprehensively and systematically review the progress of sustained and controlled release preparation technology and its application in oral drugs. Through in-depth analysis of the application effect of this technology in different types of oral drugs, it provides valuable reference for the pharmaceutical industry. The development of sustained and controlled release preparation technology is very important to improve the level of drug research and development. It can make drugs play a better therapeutic role and reduce adverse reactions. At the same time, the popularization and application of this technology also helps to promote the development of the pharmaceutical industry, create more economic value, and enhance the overall competitiveness of China ‘s pharmaceutical industry. In addition, in-depth understanding of sustained and controlled release preparation technology, for the optimization of clinical prescription.

**Methods:** This study mainly through extensive access to relevant literature at home and abroad, a comprehensive and in-depth study of sustained and controlled release preparation technology. Specific research. The research content includes its development process, from the early simple sustained release technology to the modern complex controlled release technology, and. Key technical breakthroughs and landmark achievements in each stage. At the same time, we should pay attention to the research progress of this technology, such as the research and development of new materials. The exploration of new preparation process and the construction of intelligent controlled release system.

**Results:** In recent years, significant research progress has been made in the technology of sustained and controlled release preparations. In the research and development of new materials, intelligent polymer materials and biology. Degradable materials show unique advantages, providing a new way for precise regulation and safety improvement of drug release. Innovative preparation.

**Conclusion:** The technology of sustained and controlled release preparations is of great significance for drug research and development and the development of pharmaceutical industry. It promotes the innovation and improvement of drug dosage forms. The level of drug treatment.

## 058 | Research on the integration of ergonomics and medical rehabilitation in automobile seat design

Julin Wen, Xueyan Wen

*Ganzhou polytechnic, Ganzhou, China*

**Objective:** This study aims to explore in depth the integration of ergonomics and medical rehabilitation in the design of automotive seats. Through this integration, on the one hand, the comfort of car seats can be significantly improved, allowing drivers and passengers to receive reasonable support for various parts of their bodies during long-term driving or riding, reducing fatigue. On the other hand, it can effectively protect the health of drivers and passengers, and prevent various health problems caused by long-term poor sitting posture. In addition, this innovative design method is also of great significance for promoting the development of the automotive industry. It not only meets consumers’ higher requirements for seat comfort and health, enhances the competitiveness of automotive products, but also promotes continuous innovation in design concepts and technological applications in the automotive industry, driving the entire industry towards a more humane and healthy direction.

**Methods:** This study comprehensively adopts various methods such as literature research, case analysis, and experimental research. Firstly, by extensively reviewing relevant literature at home and abroad, we aim to gain a deep understanding of the current application status of ergonomics in automotive seat design, the development of medical rehabilitation concepts and related technologies, as well as the research progress on their integration, laying a theoretical foundation for subsequent research. Secondly, using case analysis methods, select relevant cases of success and failure for in-depth analysis, summarize successful experiences and lessons learned from failures. Finally, through experimental research, the feasibility and effectiveness of the proposed design method are verified. The research mainly focuses on the specific content of the integration of ergonomics and medical rehabilitation, including seat optimization based on principles of ergonomics and seat design incorporating medical rehabilitation functions. At the same time, analyze the technical challenges, cost control, market acceptance, and other issues faced in the fusion process, and explore future development trends, such as the application of smart materials, the development of sensor technology, and the development of personalized customization.

**Results:** This study focuses on the application of the integration of ergonomics and medical rehabilitation in the design of car seats, and has achieved a series of valuable results. By deeply analyzing the principles of ergonomics, such as anthropometry and human mechanics, and combining them with medical rehabilitation theory, especially in the areas of skeletal muscle and spinal health, new ideas and methods have been provided for the design of car seats.

**Conclusion:** Looking ahead to the future, with the continuous advancement of technology, intelligent materials such as shape memory alloys and smart fabrics have broad application prospects in the deep integration of ergonomics and medical rehabilitation in automotive seats, and are expected to bring more innovation and breakthroughs to seat design. The development of sensor technology will make it possible to monitor the physical condition of drivers and passengers in real time and adjust seat functions based on feedback, further enhancing the intelligence and personalization level of seats. At the same time, the trend of personalized customization of car seats based on individual differences of drivers and passengers will become more apparent, which can better meet the health and comfort needs of different groups of people. Subsequent research can focus on these directions and continuously improve the design of car seats that integrate ergonomics and medical rehabilitation, making greater contributions to enhancing the comfort and health protection level of drivers and passengers.

## 059 | Analysis of the Changes of RBP4 Combined with Endotoxin in Elderly Patients with Coronary Heart Disease and Its Relationship with Cardiac Function

Fengmei Duan

*Department of Laboratory, People’s Hospital of Ganzi Tibetan Autonomous Prefecture of Sichuan Province, Sichuan, China*

**Objective:** To explore the changes in retinol binding protein 4 (RBP4) and endotoxin in elderly patients with coronary heart disease and their relationship with cardiac function.

**Methods:** 71 elderly patients with coronary heart disease from May 2022 to June 2024 were selected as the observation group. 67 subjects who underwent physical examination during this period were selected as the control group. The levels of RBP4, endotoxin and cardiac function between the two groups were compared. And the correlation between serum RBP4, endotoxin and cardiac function in the observation group was analyzed. ROC curves were drawn to evaluate the predictive value of RBP4 and endotoxin in major adverse cardiovascular events (MACE) in elderly patients with coronary heart disease. And multivariate Logistic regression analysis was completed.

**Results:** The RBP4 and endotoxin levels of patients with coronary heart disease in the observation group were higher than those in the control group (*P*<0.05). LVEF, SV and CI were lower than those in the control group (*P*<0.05). Pearson correlation analysis showed that the levels of RBP4 and endotoxin in patients with coronary heart disease were negatively correlated with LVEF, SV and CI of cardiac function (*P*<0.05). ROC curve results showed that the sensitivity and specificity of RBP4 combined with endotoxin in predicting MACE in elderly patients with coronary heart disease were higher than those of a single indicator (*P*<0.05). Multivariate Logistic regression analysis showed that RBP4, endotoxin, LVEF, SV and CI were risk factors for MACE in elderly patients with coronary heart disease (*P*<0.05).

**Conclusion:** RBP4 and endotoxin are highly expressed in elderly patients with coronary heart disease and are correlated with cardiac function. The combined detection of the two can predict the occurrence of MACE in patients with coronary heart disease, evaluate the progression and prognosis of the disease, and guide clinical diagnosis and treatment.

## 060 | Multifactor analysis of therapeutic effect and prognosis of video-assisted thoracoscopic surgery in elderly patients with lung cancer

Bo Zhou

*Dazhou First People’s Hospital, Dazhou, China*

**Objective:** To conduct a comprehensive investigation on the treatment efficacy and prognosis of video-assisted thoracoscopic surgery in elderly patients diagnosed with lung cancer.

**Methods:** 120 individuals were categorised into either the high-risk group (Group A) or the conventional risk categories (Group B) based on the illness category. 50 high-risk patients underwent thoracoscopic-guided anatomical pulmonary segmentectomy and systematic lymph node dissection. Finally, 70 patients in the standard risk group had thoracoscope-guided pulmonary lobectomy and meticulous lymph node dissection. This study only included patients with clinical stage I-III non-small cell lung cancer (NSCLC) who had not undergone surgery, had no N2 preoperative clinical staging, and had precise pathology results after the surgery.

**Results:** The cortical tissue credit type indicates that the proportion of AD in the high-risk group was significantly large (80%), but the proportion of SQ was nearly negligible (12%). Out of the remaining 11%, the other 8% constituted 8%. The subtype of histological categorization with the lowest proportion of lymph node metastases in Group A was determined to be AAH, AIS, MIA, LPA, APA, and PPA. This difference was highly significant, with a p-value of less than 0.01. The histological categorization serves as the fundamental basis for the reference variable. The multivariate analysis revealed that lymph node dissection involving micro-papilla and solid components in both T and lung AD cells were identified as independent risk factors for all lung AD cases associated with Group B lymph node metastases. The 3-year survival rate for individuals diagnosed with stage I lung cancer following surgery ranged from 74 to 95 percent. The 3-year survival rate of patients with stage I to III lung cancer after surgery was found to be comparable to that of patients who underwent traditional thoracotomy, with a slightly improved outcome compared to the traditional approach. This suggests that video-assisted thoracoscopic surgery may offer a superior outcome.

**Conclusion:** Lymph node dissection performed with a thoracoscope can significantly increase the rate of detecting lymph node metastases. Lung adenocarcinoma patients with T stage above T1 or with micro-papillary or solid components might benefit from lymph node dissection since it provides a more accurate pathological staging.

## 061 | Analysis of the Changes of Fasting Blood Glucose combined with Glycated Hemoglobin in Diabetic Patients and study on its Therapeutic Guidance Value

Zhi Chen

*West China Hospital of Sichuan University Mianzhu Hospital, Mianzhu People’s Hospital, Mianzhu, China*

**Objective:** To explore the changes and therapeutic guidance value of fasting plasma glucose (FPG) combined with glycated hemoglobin (HbAlc) in patients with diabetes.

**Methods:** Seventy-two diabetic patients from March 2021 to December 2022 were selected as the observation group, All were given medication combined with life guidance intervention. After 3 months of treatment, they were divided into the good prognosis group and the poor prognosis group according to the blood glucose control level. Sixty-seven healthy individuals who underwent physical examinations during the same period were selected as the control group. The levels of FPG and 2-hour postprandial blood glucose (2hPG) in each group were determined by using an automatic biochemical analyzer. The levels of HbAlc in each group were determined by latex immunoturbidimetry. The ROC curve was drawn to analyze the guiding efficacy of FPG and HbAlc in the treatment of diabetes.

**Results:** The levels of FPG, HbAlc and 2hPG in diabetic patients of the observation group before treatment were higher than those in healthy individuals undergoing physical examinations in the control group (P<0.05). For the enrolled diabetic patients, a combined intervention of medication and lifestyle guidance was adopted. After 3 months of treatment, the blood glucose of 59 patients was well controlled, and the blood glucose of 13 patients fluctuated significantly. The levels of FPG, HbAlc and 2hPG in the poor prognosis group were higher than those in the good prognosis group (P<0.05). The ROC curve showed that the sensitivity and specificity of FPG combined with HbAlc in guiding the treatment of diabetic patients were higher than those of single FPG combined with HbAlc (P<0.05).

**Conclusion:** FPG and HbAlc are highly expressed in patients with diabetes. Their expression levels can reflect the treatment prognosis of patients. The combined determination of the two indicators can enhance the therapeutic guidance value for patients.

